# Accelerating optimization of halide perovskites: two blueprints for automation

**DOI:** 10.1039/d5dd00110b

**Published:** 2025-08-25

**Authors:** Hilal Aybike Can, Daniel Anthony Jacobs, Nicolas Fürst, Christophe Ballif, Christian Michael Wolff

**Affiliations:** a École Polytechnique Fédérale de Lausanne (EPFL), Institute of Electrical and Microengineering (IEM), Photovoltaics and Thin-Film Electronics Laboratory (PV-Lab) Rue de la Maladière 71b Neuchâtel 2000 Switzerland hilal.can@epfl.ch christian.wolff@epfl.ch; b Centre d'Electronique et de Microtechnique (CSEM), Sustainable Energy Center Rue Jaquet-Droz 1 Neuchâtel 2000 Switzerland

## Abstract

The fine-tuning of halide perovskite materials for both performance and stability calls for innovative tools that streamline high-throughput experimentation. Here, we present two complementary systems designed to accelerate the development of solution-processed thin-film semiconductors. HITSTA (High-Throughput Stability Testing Apparatus) is a robust, cost-effective platform for optical characterization and accelerated aging, built around a repurposed 3D printer. It accommodates up to 49 thin-film samples, subjecting them to temperatures up to 110 °C and light intensities of 2.2 suns while continuously monitoring their absorptance and photoluminescence. ROSIE (Robotic Operating System for Ink Engineering) is a liquid-handling robot constructed from a hobbyist robotic arm and a syringe pump, enabling precise and automated ink formulation. We detail the design and operation of both systems, providing guidelines for their replication. To demonstrate their capabilities, we present a case study in which ROSIE and HITSTA are used to investigate the aging of mixed-cation, mixed-halide inorganic perovskites. Together, these systems form a powerful toolkit for accelerating the optimization of solution-processable thin-films *via* high-throughput experimentation.

## Abstract 2

The fine-tuning of halide perovskite materials for both performance and stability calls for innovative tools that streamline high-throughput experimentation.

## Graphical abstract

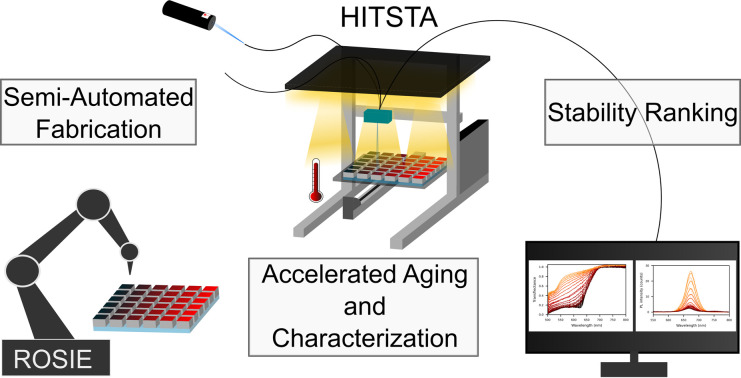

## 1 Introduction

Automated platforms enable standardization, parallelization, and cost reduction; they guarantee a higher level of reproducibility; and they liberate the scientific workforce from repetitive tasks.^1^ These benefits have been amplified by recent advances in commercially available off-the-shelf components, such as microcontrollers, robotic arms, sensors, actuators, and modular software frameworks, which have significantly accelerated the adoption of automation in research, enabling more sophisticated and scalable experimental workflows.^2^

The development of perovskite solar cells presents a compelling case for the application of high-throughput automation, given their vast compositional space and diverse fabrication routes. Perovskite solar cells have achieved remarkable status in the field of photovoltaics, emerging as one of the fastest-improving material systems to date. Power conversion efficiencies have reached 27% for single-junction devices and 34.6% for tandem devices with silicon.^3^ While a large number of research laboratories and companies are involved in the effort to optimize and commercialize perovskite photovoltaics, the still limited long-term stability of these materials remains a significant barrier. Optimism persists due to the extensive range of compositional and processing options available for fabricating perovskites in terms of compositions, additives, and processing routes (wet-chemistry or the various vapor-phase processing routes for example). This richness is also a curse however, in that it renders it impractical to explore all possibilities by evaluating them individually in solar cells made with conventional methods.^4^ Moreover, the interdependence between processing parameters suggests that many should be co-optimized rather than optimized independently, as is often the case in conventional experimental approaches. However, co-optimization demands a substantial number of experiments to identify optimal conditions even with sophisticated machine-learning approaches.^5,6^ There is, therefore, a need for efficient, high-throughput strategies that can systematically explore and refine these parameters to accelerate the discovery of stable and high-performance perovskite materials and devices.

To quantify the potential impact of automation on perovskite research, we conducted an informal study amongst colleagues in our laboratory to address the question of how much time is spent on the various steps of a typical optimization experiment. Based on this survey, we estimate that the time required to execute a relatively standard perovskite experiment aimed at optimizing device efficiency is divided roughly as follows: planning (2.5 h, 17%), preparation of solutions (1.25 h, 8%), substrate + preparation of the first contact layer (1 h, 5%), perovskite fabrication (3 h, 19%), remaining layers (5 h 37%), and evaluation with IV measurements (2 h, 14%) (Fig. S12). These estimates reflect the total duration of each step rather than the work required (*e.g.*, atomic layer deposition is time-intensive but largely automated). While approximate, these figures highlight the fact that no single task dominates the optimization of perovskite devices at the level of device efficiency. Efforts to accelerate such optimization must therefore address multiple experimental tasks in parallel to achieve substantial time savings. The situation changes significantly when optimization targets film stability as well as performance however, since aging experiments typically require hundreds to thousands of hours at the evaluation stage. This dwarfs the time needed for device fabrication, and means that aging must be either highly parallelized (difficult due the infrastructure requirement of having multiple large-area aging chambers) or that an accelerated evaluation must be achieved by other means.

As noted above, efforts to accelerate perovskite optimization at the level of performance must target several steps simultaneously to be effective. A common approach is to abbreviate the process of cell fabrication by screening proxies for full devices, *i.e.* the perovskite in some form without the additional contact layers comprising a full device. For example, in separate studies Chen *et al.*^7^ and Higgins *et al.*^8^ bypassed cell-fabrication entirely by conducting photoluminescence (PL) and absorption measurements on precipitated perovskite crystals, utilizing commercial liquid-handling robots for automated solution preparation. Similarly, Li *et al.* used a liquid handling robot and a high-throughput automated approach based on inverse temperature crystallization (ITC) to identify and optimize synthesis conditions for perovskite single-crystals.^9^ In a follow-up study, Zhao *et al.* employed an extension of the same robotic platform to fabricate perovskite films *via* drop-casting, demonstrating rapid and reproducible film formation.^10^ Such approaches, based on the evaluation of highly simplified proxies, could be valuable to the extent that they reflect the performance and stability of realistic devices. However, as noted by Zhao *et al.*, the deposition method (drop-casting *versus* spin-coating) was the most important variable dictating material quality among all the parameters tested, including compositional variables.^10^ This supports the consensus of myriad studies that intricate control of the perovskite crystallization process is key to obtaining performant and stable films,^11^ and suggests that, to be considered reliable, comparative evaluations must be made on thin-films crystallized *via* a pathway applicable to functional devices.

Instead of making proxies, acceleration can be achieved by incorporating robotic assistance into the fabrication workflow. Zhang *et al.* introduced an automated platform combining a commercial multipurpose robotic arm (SCARA) and a spin-coating robot (Spinbot) for engineering solution-processed thin films.^12^ Cakan *et al.* also developed an automated production-line for spin-coating, including in-line photoluminescence characterization.^13^ Both studies represent impressive achievements in lab-scale automation. However, while these automated approaches significantly improve fabrication speed and experimental repeatability, they primarily focus on material screening and rapid characterization rather than long-term stability assessments. Understanding and quantifying the degradation mechanisms of perovskite materials remains a critical challenge for thin-film devices made thereof, as device stability is a key bottleneck for commercial deployment. Therefore, developing automation strategies that incorporate some form of accelerated aging and degradation monitoring is essential for advancing the long-term reliability of perovskite solar cells.

Building upon these conclusions, in the following we introduce HITSTA (High-Throughput Stability Testing Apparatus), a system designed to perform high-throughput optical characterization and stability assessment of perovskite materials under controlled heat and light-stress. By focusing on purely optical means of characterization (photoluminescence and optical absorption) HITSTA enables the assessment of both performance and intrinsic stability for partial-device proxies. However, this choice remains one for the user to make: the system is equally capable of evaluating samples consisting of just a single film on glass on the one hand, and full device-stacks including all selective contact layers and electrodes on the other. Another advantage of optical characterization is that it allows the measurement of “whole-film” averaged properties, if designed appropriately, whereas electrical measurements such as IV-characteristics and MPP tracking are vulnerable to the presence of even a single microscopic contaminant or weak spot (*e.g.*, electrical shunt paths).

Our approach with HITSTA is philosophically similar to that of Keesey *et al.*, who developed an open-source aging chamber for high-throughput, semi-automated stability measurements, based on the idea that optical changes in the visible spectrum can serve as a viable non-invasive *in situ* monitoring technique.^14^ The white-light measurements implemented in the HITSTA platform offer richer spectral information, as exemplified in studies like that of Merdasa *et al.*,^15^ albeit at the cost of increased measurement time and system complexity. In addition to white-light measurements, HITSTA can periodically perform photoluminescence (PL) measurements, enabling the estimation of the open-circuit voltage (*V*_oc_).^16^ Changes in PL during degradation provide both a second means for evaluating sample stability, and potentially deeper insights concerning the role of processes like halide segregation and their relationship with different degradation modes.

As in the studies above,^5–7,9,14^ we identified the need for a robotic liquid-handling system to perform more thorough explorations of the vast compositional space of halide perovskites. Although the solution-preparation step takes only a modest fraction of the total time in a typical optimization experiment (8% according to our survey), this number quickly balloons when there are many compositional variations or additives to be explored. Operator error also becomes an increasing concern with complex mixing tasks (*cf.*Fig. 3). While commercial robotic systems are available to perform these tasks, they may not be feasible for every laboratory due to budgetary constraints. Moreover, challenges such as limited customization, restricted software access, and difficulties in addressing technical or software issues further complicate the adoption of commercial systems. We therefore also share here our designs for ROSIE (Robotic Operating System for Ink Engineering), a compact and affordable liquid handling robot based on a consumer electronics robotic arm^17^ and an Arduino-controlled syringe pump.

## 2 Methods

### 2.1 HITSTA: system layout

**Fig. 1:**
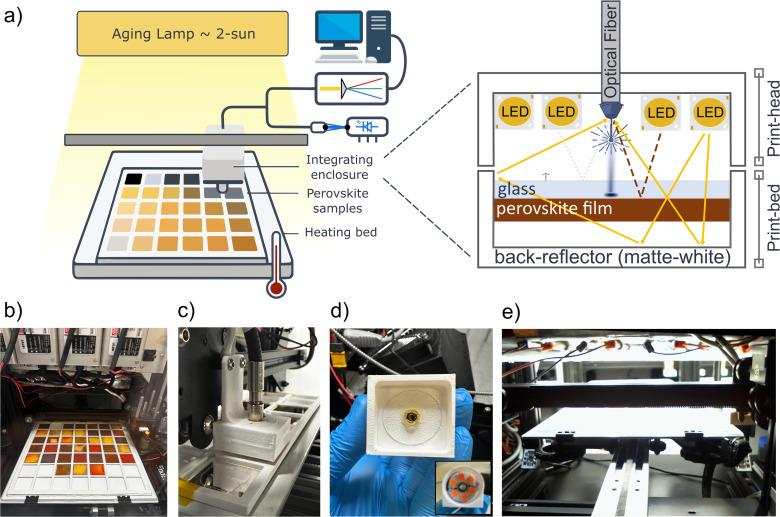
(a) Schematic of HITSTA indicating its three primary components: the modified print-bed, the modified print-head and the aging lamp. Inset shows the “integrating enclosure” comprising the sample backplate and the modified print-head, which contains LEDs for broadband illumination and an optical fiber bundle with fibers to the spectrometer and a blue laser. (b–e) Photographs of (b) the modified print-bed, (c) the modified print-head (measurement head), (d) the underside of the measurement showing the optical fiber and the LEDs which lie behind an annular diffuser, and (e) the print-bed in position under the aging lamp.

HITSTA is designed for high-throughput optical characterization and thermally accelerated aging^18^ of perovskite or other thin-film semiconductors. It repurposes an entry-level 3D printer to automate sample manipulation, with the printer's movable head enabling multi-sample measurement, and its heated bed being repurposed to thermally accelerate the aging process (Fig. 1b). With a sample-holder designed for the popular format of 25 × 25 mm glass substrates, the system can accommodate up to 49 samples simultaneously, or potentially many more samples in a smaller format. The sample holder is mounted directly onto the printer bed, which can be heated from room temperature to 110 °C for controlled thermally-accelerated aging. Meanwhile, the modified print-head integrates an optical fiber bundle, a reflective enclosure (an imperfect miniature Ulbricht enclosure), and broadband white LEDs (Fig. 1c and d). The fiber bundle directs light from inside the reflective enclosure to an Ossila USB spectrometer (spectral range: 320 nm to 1050 nm), and a blue fiber-coupled laser (445 nm, Insaneware) is included to illuminate samples at high intensity for photoluminescence measurements. The spectrum of the white LEDs as measured within the system is shown in Fig. S5a.

In ‘one-shot’ mode, HITSTA can screen up to 49 samples in a timeframe of minutes based on optical properties (approx. 10 s per sample depending on integration times), a process that would typically take several hours using conventional UV-vis and photoluminescence tools. In this mode, HITSTA enables a rapid feedback loop for processes that can be evaluated purely by optical means, such as bandgap tuning or maximizing PL yield. However, the primary purpose of HITSTA is to enable accelerated evaluations of sample stability. To this end the system includes a high-intensity aging lamp with automated shuttling of samples between measurement (Fig. 1b) and aging (Fig. 1e) positions. This enables monitoring the evolution of PL and absorptance after repeated cycles of controlled light exposure, and at elevated temperatures up to 110 °C. The acceleration offered by HITSTA relative to a more standard workflow arises from its high-capacity sample plate, intense aging lamp, and heated bed, with further time-savings possible when evaluating partial-device stacks. A video of the system in operation is included as Movie S2.

### 2.2 HITSTA: optical “Transflectance” measurements

**Fig. 2:**
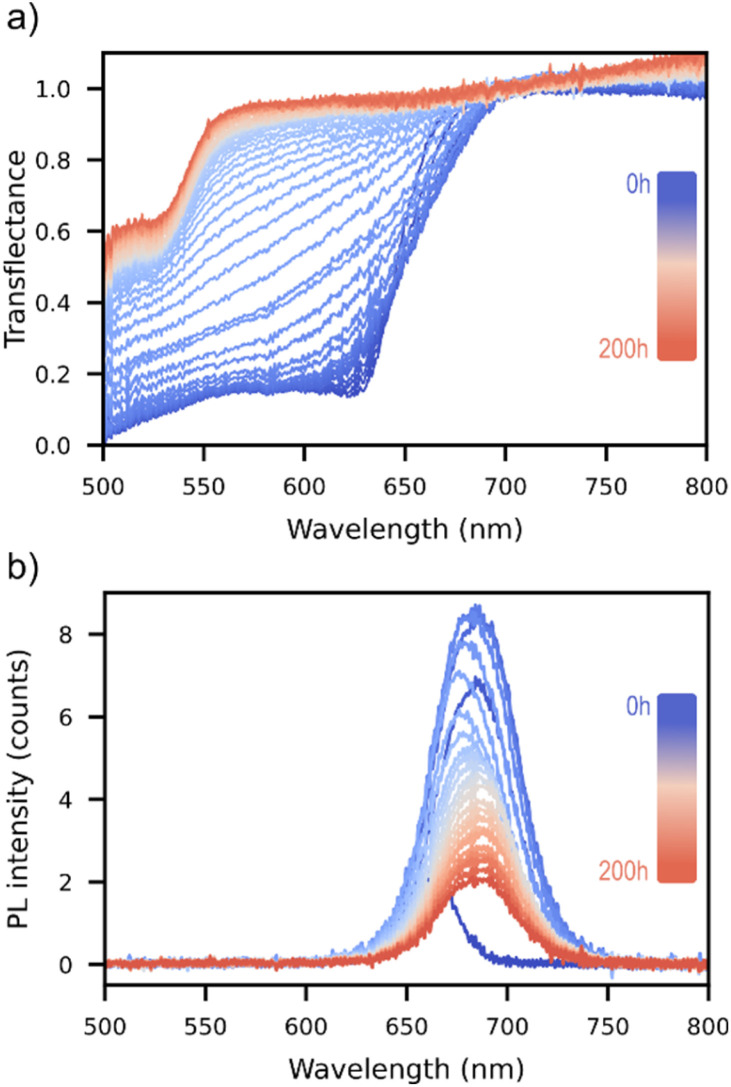
(a) Transflectance and (b) PL measurements taken by HITSTA on two CsPbI_2_Br samples in their initial states (as fabricated) and as a function of time spent light-soaking under HITSTA's aging lamp (approx. 2 sun) at 80 °C (measurements acquired at 40 °C). In (a) the disappearance of the perovskite band-edge at ≈650 nm is followed by the growth of a new band-edge at ≈550 nm, whilst the PL spectrum in (b) is seen to red-shift and decrease continuously in intensity.

To monitor optical absorption, light from a broadband LED array attached to the measurement head is directed through samples from the glass-side. The matte-reflective backplate mounted on the printer bed returns any transmitted light through the sample towards an optical fiber in the middle of the measurement head (Fig. 1d). The collected light therefore comes predominantly from rays that are, in sequence: (1) transmitted through the sample (2) reflected from the backplate, and (3) transmitted through the sample again. Smaller contributions stem from rays that are scattered multiple times from the matte-reflective surfaces, and from specular and diffuse reflections at the other interfaces (glass–air, glass–perovskite, and perovskite–air, *cf.*Fig. 1a inset). Spectra such as the one shown in Fig. 2a are obtained by dividing this signal by that of a “null” measurement with no sample in place, *i.e.* expressing light collection in the enclosure relative to that of a perfectly transparent sample.

Evidently the measurement described above differs from a strict transmittance or reflectance measurement. We use the term “transflectance” to indicate that both transmittance and reflectance contribute to the measured signal, as in the configuration employed by Merdasa *et al.*^15^ After a few design iterations, we found the most satisfactory results were obtained by including reflective baffles around the fiber head that mimic an Ulbricht enclosure (Fig. 1d). The Ulbricht enclosure significantly reduces the sensitivity of transflectance measurements to scattering from rough samples. The limitations of this partially open enclosure only become apparent when measuring highly textured films, which can occasionally reflect light into the collection cone more effectively than the enclosure walls, resulting in transflectance values exceeding unity. Nevertheless, even in such cases, meaningful data can still be extracted by analyzing features such as the slope of the transflectance spectrum near the band edge and its evolution over time. As shown in Fig. 2a and S7a, the transflectance provides clear information regarding the band-edge (its position and steepness), as well as indications of absorption above and below the bandgap. In the example of Fig. 2a, the transflectance spectrum of the fresh sample displays a sharp band edge, characteristic of its initial photoactive state. Over time, this sharp band edge becomes less defined as the sample ages, whilst gradually a new onset appears at a shorter wavelength. This example reveals that by measuring over a broad wavelength range HITSTA is not only able to monitor the photoactive perovskite phase, with bandgaps typically in the range of 1.5–2 eV (*ca.* 650–800 nm), but also the appearance of secondary phases. We revisit the 550 nm absorption edge in Fig. 5, but note here that the ability to optically monitor multiple phases is especially valuable for inorganic perovskite solar cells, where phase instability limits long-term performance.

### 2.3 HITSTA: photoluminescence measurements

HITSTA's current design incorporates a blue laser (445 nm, maximum power 700 mW, Insaneware), chosen for its applicability to high-bandgap perovskite materials. The laser light is coupled into an optical fiber (Thorlabs-FT600EMT) extending to the measurement head, which is typically positioned at the center of the sample for single-shot acquisitions. Multi-point measurements could, in principle, assess film uniformity, but off-center positioning of the measurement head may reduce collection efficiency due to imperfect closure of the two-part Ulbricht enclosure. The intensity of the laser spot was measured at 39 mW spread over a spot size of 0.7 ± 0.02 mm (2.5 W cm^−2^). In experiments integration times of 1000–5000 ms are typically used to obtain sufficiently high signal-to-noise ratios. A long-pass filter (Thorlabs-FGL495) is positioned before the entrance of the spectrometer to remove the high-intensity laser line, which sets the lower limit of measurement for both the PL and white-light modes at 495 nm.

Fig. 2b shows an example set of photoluminescence spectra measured with HITSTA on a pristine CsPbI_2_Br [+8% FACl] sample and as a function of time spent aging under the aging lamp at 80 °C. All HITSTA measurements reported in this paper were performed with 40 °C as a reference point,^19^*i.e.* samples were cooled from the aging temperature before each round of optical measurement.

### 2.4 HITSTA: accelerated aging

To age samples, HITSTA houses an aging lamp (V-TAC 200 W, 16500 lm LED Floodlight) capable of delivering up to 2.2 suns equivalent illumination depending on the sample bandgap (see Fig. S8c). Samples are moved between the measurement position (under the modified print-head) and the aging position (under the lamp) using the print-bed *y*-axis. The Ender 3's stock print-bed heats the sample holder to a maximum of 110 °C, a temperature sufficient to meaningfully accelerate perovskite degradation.^18^ Sample temperatures track the bed temperature to within a few degrees even when separated by glass spacers. A sheet of plexiglass is positioned in front of the aging LEDs to ensure uniform light distribution across the samples, and mirrors around the aging lamp are placed to further increase uniformity. Feedback in the printer-bed ensures that the bed temperature remains at the setpoint even under the high-intensity aging lamp, although heating from the latter does impose a minimum aging temperature of 54 °C under maximum illumination. In its current form, the uniformity of illumination under HITSTA's aging lamp depends on the sample arrangement: with 37 optimally positioned samples, intensity variation is below 10%, whereas using the full capacity of 47 samples increases variation to 28% (Fig. S8d). To correct for spatial variation in light intensity, an appropriate aging model — *e.g.* assuming a linear degradation rate with light intensity^20^ — could be applied to normalize aging times across sample positions. For the showcase experiment disclosed here we simply utilized the 37 sample positions with <10% non-uniformity. Better uniformity could be obtained with a larger aging lamp relative to the sample bed, or a thicker diffuser (at the expense of some intensity).

### 2.5 ROSIE: robotic operating system for ink engineering

In order to enable in-depth exploration of the chemical parameter space, a robotic liquid-handling system (ROSIE) was built to aid in preparing perovskite solutions. At the heart of ROSIE is a ‘uArm Swift Pro’ robotic arm, paired with an Arduino-controlled syringe pump for precise pipetting. To enable the robotic arm to operate a pipette, we 3D-printed the holder designed by OpenLH.^17^ Meanwhile the pipette tube was taken from a disassembled manual pipette. The syringe pump achieves a relative error of 1.5% when handling 45 μL of solution (Fig. S2d), which notably surpasses the 3% relative error observed with the lab's commercial manual pipettes (20–200 μL range) for 100 μL volumes. In its current state ROSIE is capable of mixing solutions from up to eight different precursors and generating 96 distinct solutions in a well plate (polypropylene) within approximately one hour. A video of ROSIE in operation is included as Movie S1.

### 2.6 Showcase experiment: ink preparation

**Fig. 3:**
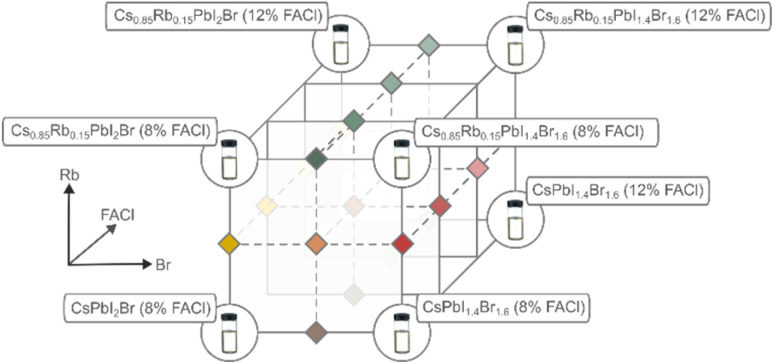
A total of 36 perovskite compositions were derived from mixtures of eight stock solutions to form a 3-dimensional space in the parameters of Cs : Rb ratio, I : Br ratio, and FACl molarity.

Wide-bandgap (1.8–2.0 eV) inorganic perovskite absorbers were synthesized with eight stock solutions using the ROSIE platform, as indicated schematically in Fig. 3. The precursor solutions were prepared using 0.8 M PbI_2_ (TCI, >99.999%, trace metals basis), PbBr_2_ (TCI, >99.99%), CsI (Alfa Aesar, 99.9%, metal basis), FACl (Greatcellsolar, 99.99%), and RbI (Sigma-Aldrich, 99.9%, trace metals basis), dissolved in dimethyl sulfoxide (DMSO, Sigma-Aldrich, anhyd.). Mixtures were made into a 96 well plate (polypropylene, Axygen). The nominal compositions of each combination tested in the experiment are listed in Table S1 of the SI. After completion, the well plate of solution mixtures was transferred from the N_2_ glovebox housing ROSIE to the spin-coating glovebox in an airtight bag.

### 2.7 Showcase experiment: sample fabrication

Substrates of 2.5 × 2.5 mm glass (Tking Glass, China) were sonicated in a 2% Hellmanex solution for 40 minutes, followed by sonication for a further 40 minutes in de-ionized water and finally isopropyl alcohol. After drying with compressed air, the substrates were subjected to UV-ozone for 30 minutes, and then immediately transferred to the N_2_ glovebox for spin-coating (commencing less than 15 minutes after the treatment). For the inorganic perovskite layers, 100 μL of inorganic perovskite precursor solution was manually spin-coated onto the glass substrates at 3000 rpm for 70 s in a N_2_ glovebox. A chiller was used maintain the atmospheric temperature in the glovebox between 21–23 °C during this spin-coating step. The films were first annealed at 42 °C until they turned to a uniform dark red-brown. Since each composition in the well plate was different, the annealing time varied from 40 seconds to 1 minute depending on the film. Immediately after the first-stage annealing, samples were annealed again on a second hot-plate inside the spin-coating N_2_ glovebox at 180 °C for 5 minutes.^21^ All 36 samples were prepared in a continuous spin-coating session (duration approx. 90 min from the start of perovskite coating), before being transferred in an airtight box to a separate N_2_ glovebox containing the HITSTA platform.

### 2.8 XRD measurements

XRD measurements were performed in a Bruker D8 Discovery diffractometer using Cu K-α radiation in Bragg–Brentano geometry. Scans in 2*θ* were performed within the 5–50° range with a resolution of 0.03°. A gas-tight XRD sample chamber equipped with a 1 mm thick polyether ether ketone (PEEK) dome was used to minimize the effects of atmospheric exposure.^22^

## 3 Results and discussion

### 3.1 Description of 3-parameter showcase experiment

To demonstrate the capabilities of HITSTA and ROSIE in high-throughput aging and solution preparation, we designed an experiment to explore the compositional space of inorganic perovskites. For this experiment it was decided to vary simultaneously the cation ratio (Cs to Rb)^23^ and the halide ratio (I to Br), while incorporating FACl as an additive to modulate crystallization at concentrations ranging from 8% to 12% (mol% to Pb).^21^

For this showcase experiment, we elected to evaluate perovskite films spin-coated on glass, *i.e.* to work with simplified proxies of a full device stack. This represents a departure from the most conservative strategy for sample evaluation, which would be to include all the layers of a complete solar cell, up to and including encapsulation layers, to address a maximal number of potentially relevant degradation modes at the same time. However, such “full-stack optimization” comes at a considerably higher cost in throughput compared to evaluating perovskite films on glass. It is furthermore not unreasonable to expect that if candidate film A shows vastly superior stability to that of film B when evaluated as bare films on a substrate, that the same is likely to hold true at the level of complete devices. However, for the moment this remains merely a likely conjecture that requires further study to evaluate its merits as an optimization strategy. As noted in the introduction, HITSTA can be used to evaluate cells at any level of completion from bare films on glass to complete cell stacks. We choose films on glass here to demonstrate a rapid mode of sample evaluation that is enabled by the use of purely optical techniques.

Using our liquid-handling robot ROSIE a total of 36 compositions in a parameter grid of 3 × 3 × 4 (Fig. 3) were prepared in a 96-well plate, starting from 8 stock solutions. Films were fabricated by spin-coating the mixtures onto 25 × 25 mm glass substrates, then transferred to a nitrogen glovebox housing HITSTA for an initial optical characterization. Subsequently, periodic measurements and automated aging were conducted for a duration of 200 hours under the lamp. The aging process was carried out at a controlled temperature of 80 °C under full power aging intensity (approx. 2.2 suns for bandgaps of 1.9–2 eV, see Fig. S8c). White light and photoluminescence (PL) measurements were recorded every 2 minutes at the beginning of the aging process, with pre-programmed intervals increasing to 6 hours as the experiment progressed and degradation slowed.

### 3.2 Quantifying degradation with optical measurements

**Fig. 4:**
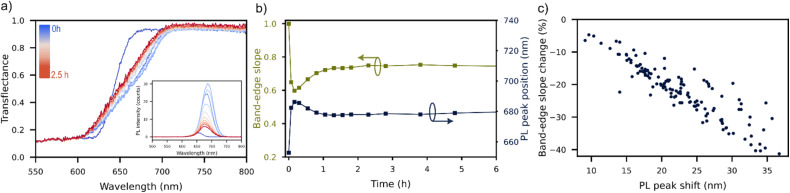
Short-term aging behaviour of inorganic perovskite samples measured and aged in HITSTA. (a) Evolution of transflectance data in the first 10 measurement-aging cycles, with inset showing the PL data of the same sample. (b) Time series for a single sample showing the co-variance of the band-edge slope and PL peak position over the first six hours of aging. (c) Data points taken from all samples in the experiment (with measurable PL peaks) over the first six hours of aging, demonstrating a robust correlation between changes in the transflectance and PL.

All samples in this experiment exhibited qualitatively similar aging behaviour unfolding across two distinct timescales: a rapid phase lasting minutes to hours, followed by a slower degradation process spanning tens to hundreds of hours. In particular the perovskite band-edge in transflectance was observed to “soften” over the first few minutes (Fig. 4a), followed by more gradual changes over the following hours. Simultaneous measurements of the sample PL show that this rapid decrease in slope at the band-edge coincides with a red-shift in the PL peak position (Fig. 4b), a process typically attributed to halide segregation.^24^ Plotting the shift in PL peak position and change in steepness over the first 6 h of measurement for each sample results in Fig. 4c, demonstrating a clear correlation between the two quantities. It can be concluded that the early time-evolution of these inorganic samples is dominated by halide segregation in both PL and transflectance measurements.

**Fig. 5:**
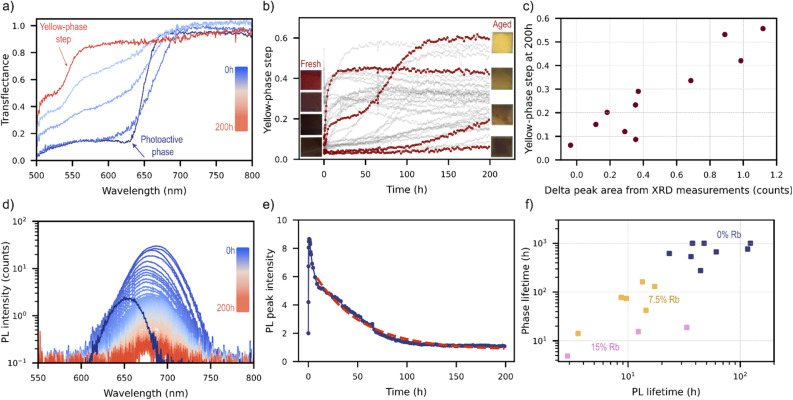
Long-term aging behaviour of inorganic perovskite samples (Rb_*x*_Cs_1−*x*_PbI_*y*_Br_3−*y*_ films on glass) measured and aged in HITSTA at 80 °C and 2.2 suns. (a) Evolution of the transflectance data showing the decay of the perovskite band-edge and the growth of a new band-edge at approximately 550 nm. (b) Time-series data showing the growth of this band-edge for all samples in the experiment (here computed as Trf(540 nm)–Trf(510 nm) with Trf denoting the transflectance). Insets are photographs of selected samples shown in the same order (top-bottom) both before and after aging. (c) Correlation between the size of the transflectance step at 550 nm and the area of the XRD peak at 2*θ* = 10.0°. (d) Long-term evolution of PL spectra showing decay to a small fraction of the initial intensity. (e) Plot of the PL intensity (obtained by Gaussian fitting to the spectra in (d)) over time with a stretched exponential fit. (f) Relationship between the fitted “lifetime” obtained from PL intensity in (e) and the corresponding lifetime obtained from making exponential fits to the curves in (b), termed here the “phase lifetime”.

After the brief halide segregation phase (<1 h), the transflectance spectra temporarily stabilize before undergoing further band-edge softening over tens to hundreds of hours under the lamp (Fig. 5a). On this longer timescale there is the notable formation of a new shoulder in the transflectance spectrum at a shorter wavelength of approximately 550 nm. The size of this step in the transflectance at 550 nm is plotted over time in Fig. 5b, with insets showing the visual appearance of samples before and after aging. Samples exhibiting stronger absorption steps at 550 nm notably displayed a yellow coloration after aging (Fig. 5b insets). We posit that this degraded state, labelled here as the “yellow-phase” for simplicity, is a segregated mixture containing δ-Rb_*x*_Cs_1−*x*_PbI_3_, γ-Rb_*x*_Cs_1−*x*_PbI_3,_ γ-Rb_*x*_Cs_1−*x*_PbBr_3_, and the parent material (nominally γ-Rb_*x*_Cs_1−*x*_I_*y*_Br_3−*y*_). XRD measurements taken on degraded samples show a characteristic peak at 2*θ* = 10.0° (Fig. S10a), which is our principal evidence for the presence of δ-Rb_*x*_Cs_1−*x*_PbI_3_ in the degraded films.^21^ Interestingly, the size of the peak at 2*θ* = 10.0° is found to correlate with the size of the absorption step at 550 nm as shown in Fig. 5c, however, this wavelength differs from the bandgap of δ-Rb_*x*_Cs_1−*x*_PbI_3_ at 2.84 eV (approx. 440 nm).^25^ Instead, the shorter-wavelength step at 550 nm coincides with the bandgap of γ-Rb_*x*_Cs_1−*x*_PbBr_3_, which may form as a segregated phase during degradation of the parent material. Signs of a γ-Rb_*x*_Cs_1−*x*_PbI_3_ component were also observed in some films in the form of a red-shifted band-edge following aging at wavelengths corresponding to bandgaps of approximately 1.7 eV (Fig. S10b).

Over tens to hundreds of hours, changes in PL are characterized by gradual darkening with minimal alteration in spectral shape (Fig. 5d). By fitting exponential or stretched-exponential models to both yellow-phase growth (Fig. 5b) and PL peak decay (Fig. 5e), we extract two distinct ‘stability lifetimes’ for most samples. Lifetimes for phase-change could not be assigned to samples that initially contained a large yellow-phase fraction or exhibited an extremely slow transition, as the fitting process becomes degenerate in these cases. Similarly, PL lifetimes were only assigned to samples with measurable PL intensity, a criterion that largely excludes those with high initial yellow-phase content.

Among samples where both lifetimes could be determined (*τ*_phase_ and *τ*_PL_), the two appear monotonically related (Fig. 5f). In principle, we would expect PL brightness near the perovskite bandgap to decay at least as quickly as the yellow-phase grows—that is, PL should decrease (at minimum) in proportion to the remaining perovskite material. Fig. 5f confirms that this inequality (*τ*_PL_ < *τ*_phase_) holds for most data points. Moreover, the observed monotonic relationship (log *τ*_PL_ ≈ log 0.7*τ*_phase_), although approximate, suggests that phase-change may be the dominant process driving electronic degradation in these perovskite samples. That is, within this dataset, we find no evidence of additional hidden parameters affecting the relationship between *τ*_PL_ and *τ*_phase_.

To summarize, our observations reveal two distinct stages in the degradation of mixed-halide inorganic perovskite samples: one associated with halide segregation on the timescale of minutes-hours, and the slower process pf phase degradation on the timescale of tens to hundreds of hours. The interpretation of these phenomena was enabled by HITSTA's complementary measurement of transflectance and PL on each sample as a function of aging time, whilst functional relationships could be inferred by gathering measurements on many samples simultaneously.

### 3.3 Analysis of compositional trends

**Fig. 6:**
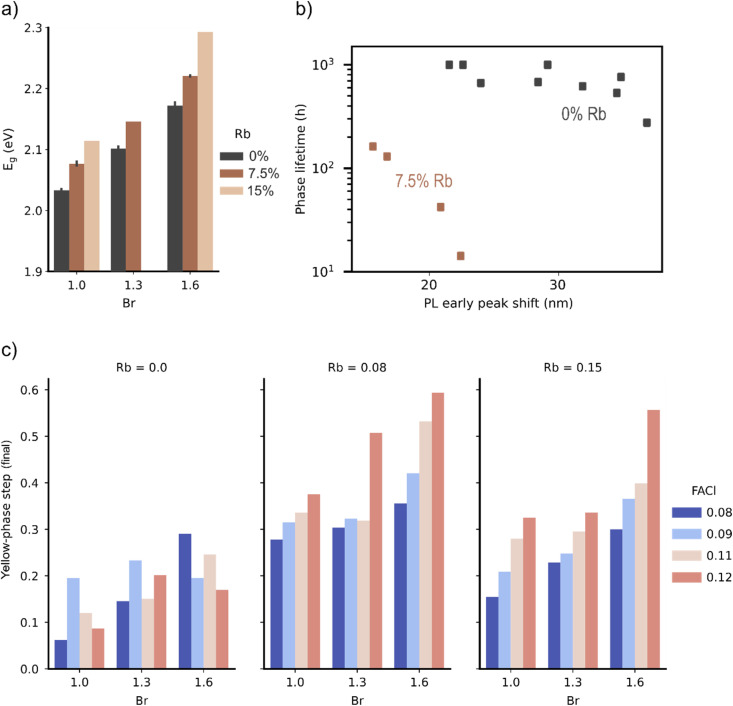
(a) Bandgap estimated from transflectance data as a function of compositional variables. (b) Scatter plot of the phase lifetime and PL redshift showing groupings according to Rb fraction. (c) Effect of compositional variables on the size of the delta-step in transflectance following 200 h of accelerated light-soaking (≈2 suns at 80 °C).

Our showcase experiment revealed systematic trends in all three of the compositional variables, namely the I : Br ratio, the Cs : Rb ratio, and the FACl content. The incorporation of Rb into Cs-containing perovskites was introduced in ref. 21 where it was shown that substitution of Cs by Rb increases the perovskite bandgap up to a threshold determined by the I : Br ratio. The data gathered by HITSTA before aging corroborates this claim, with bandgaps estimated from the transflectance measurements showing a systematic trend in both Br and Rb content (Fig. 6a). PL proved less reliable for initial bandgap determination on this sample-set due to the absence of measurable luminescence in some films (typically those with a large fraction of the yellow phase in the as-fabricated state), and the presence of substantial segregation in others (either present in the as-fabricated films or developing during the initial measurement rounds under LED and laser illumination).

**Fig. 7:**
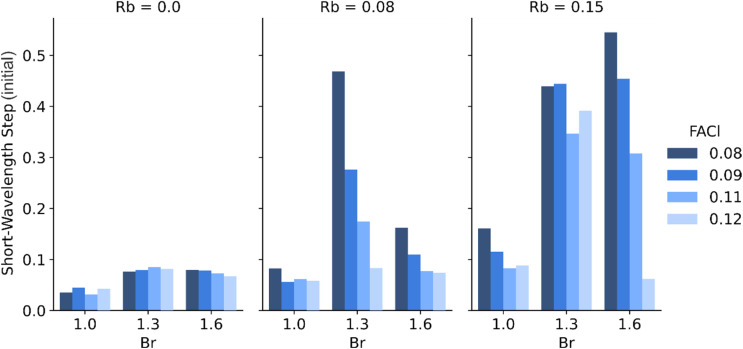
Initial short-wavelength step size as a function of Br content for three Rb levels (0.0, 0.08, 0.15) and varying FACI ratios. Step size (and hence yellow-phase content in the film) increases with Rb and FACI, particularly at intermediate Br compositions.

As mentioned, not all compositions tested in this experiment yielded films consisting of primarily the desired photoactive phase. This was particularly true at the highest Rb fraction of 15%, where only a few of the tested ink mixtures yielded dark films following the second stage of annealing (Fig. 7). We observed an interesting dependence on the Br fraction, wherein the highest number of yellow-phase films occurred at Br fraction 43% (Rb_*x*_Cs_1−*x*_I_1.6_Br_1.4_), suggesting an “island of instability” at these intermediate Br values. We furthermore observed a stabilizing effect of FACl addition which had a systematic effect in terms of reducing the yellow-phase fraction at the intermediate Br fraction of 43% with 8% Rb, and at 53% Br with 15% Rb.

According to Wang *et al.*,^21^ Rb incorporation serves primarily to suppress halide segregation and enhance operational stability. In our sample set it was indeed observed that Rb addition up to 7.5% suppressed halide segregation by reducing the redshift of PL peak position during aging (Fig. 6b). Here we quantified this shift (“PL early peak shift” in the figure) by measuring the red-shift of the PL peak position after 30 min of aging (*cf.*Fig. 4b) relative to its initial position. Ref. 18 observed apparently complete suppression of halide segregation at 15% Rb fraction, however in our samples with 15% Rb the PL intensity was diminished to below the noise level, rendering a comparison impossible. In addition to this undesirable PL quenching, Rb addition was observed to de-stabilize the perovskite phase in our experiment, precipitating a faster conversion to the yellow-phase under accelerated aging (Fig. 6b).

The discrepancy between our stability findings and those reported by Wang *et al.* may be attributed to several key differences in sample fabrication. First, our samples were prepared on glass substrates instead of on ITO/NiO_*x*_ as in Wang *et al.* The choice of substrate is well-known to affect crystallization in a way that can dramatically change phase-stability, as vividly illustrated when working with partial-area ITO substrates containing glass-only regions (Fig. S9c). An additional clue is provided by plotting the size of the yellow-phase step in the transflectance data after 200 h of aging as shown in Fig. 6c. Here we see that Rb-addition generally increases the amount of yellow-phase present after aging in proportion to the FACl content (*i.e.* additional FACl is detrimental to the phase stability). This points to a lower optimum value for the crystallization additive than the minimum molar percentage of 8% (to Pb) used here. However, this systematic trend with FACl concentration (Fig. 6c) disappears in samples without Rb. This complex interdependence between compositional variables underscores the necessity for multi-dimensional, high-throughput optimization approaches when developing stable perovskite formulations, jointly with the underlying layers.

### 3.4 ROSIE: status and future iterations

Despite the fact that commercial liquid-handling systems are well-established in the life sciences, they remain rare in perovskite research, appearing mainly in work from automation-focused labs.^7,9,10,12,13^ This may reflect either (1) a presumed inability to make use of large solution arrays in the absence of higher levels of automation, or (2) concerns over cost-benefit. In the case of (1), our showcase experiment demonstrates that a robot like ROSIE can facilitate sophisticated chemical trials even within a manual spin-coating workflow. While preparing 36 inks from eight stocks is conceivable by hand, the risk of error or time burden is clearly a deterrent for such efforts. As for cost, commercial robots typically range from €13k–70k, which is comparable to that of many other laboratory assets such as characterization equipment and gloveboxes. Since research in photovoltaics benefits from access to a large number of tools, PIs are typically faced with choices of prioritization, such as whether to purchase a liquid handling robot or an additional glovebox plus spin-coater, or perhaps an ellipsometer.

By contrast, ROSIE's estimated cost of €1300 in components and raw materials makes it highly accessible. To support adoption, we provide a full parts list with suppliers and prices in Table S3, as well as CAD files for custom parts on GitHub (https://github.com/hilalaybikecan/AutoPVLab.git). An assembly guide is included in the SI.

ROSIE offers several additional advantages: its open design supports easy reconfiguration (*e.g.* vial sizes, pipette volumes); it uses inexpensive single-use pipettes; it is user-serviceable without proprietary support; and its Python control scripts are easily customizable. The current setup could furthermore serve as the liquid-dispensing component in a fully automated spin-coating or drop-casting line—potentially enhancing reproducibility across labs, a long-standing challenge in perovskite research. Full automation would however require integration with a second robotic system for substrate handling, and faster sample throughput would ideally be paired with high-throughput tools like HITSTA for evaluation.

The main limitations of the current ROSIE system are its speed and lack of pipette pickup sensing. A typical 36-ink run takes 30 to 90 minutes depending on matrix sparsity. While this is acceptable for low-volatility solvents like DMF and DMSO, it could be problematic for more volatile inks due to evaporation. Speed is limited by the syringe pump's stepper motor, which currently runs at a slow speed to avoid overheating. Improvements might include active cooling or reduced plunger friction. Higher flow rates, possibly *via* larger syringes, would also be needed for fast anti-solvent delivery in spin-coating applications.

Pipette pickup currently depends on precise, hard-coded positions, making the system sensitive to small misalignments. This is mitigated through careful setup and frequent homing, but could be improved with tip-detection *via* machine vision or other sensing.

Lastly, ROSIE's air-cushioned syringe was optimized for DMF and DMSO. With lower surface-tension solvents like isopropanol, dripping can occur. Future development may include pre-wetting cycles, hydrophobic pipette tips, or slight syringe retraction during arm movement to improve compatibility with volatile inks.

### 3.5 HITSTA: status and future iterations

By contrast with ROSIE, HITSTA as presented here provides a combination of capabilities that, to our knowledge, are not commercially available in any form at this time. While several research platforms have incorporated optical methods for high-throughput perovskite characterization, such as the PASCAL system which integrates robotic synthesis and optical readout,^13^ few address long-term stability under combined heat and light stress. In the absence of validated models linking early-time optical signatures to long-term degradation, we believe that such data cannot replace empirical stability testing. If they are to be developed, predictive models for long-term stability will require large, compositionally diverse datasets generated on platforms like HITSTA, which combine long-term stressing with automated, high-throughput measurement.

The closest published analogue to HITSTA was reported by Keesey *et al.*, who disclosed several designs for chambers combining heat and light-stress with *in situ* optical monitoring *via* colour-corrected photography.^14^ This design allows for the simultaneous monitoring of many samples under continuous aging stress. Meanwhile HITSTA trades continuous *in situ* tracking for an approach based on aging-measurement cycles that enables acquisition of rich optical data. This in turn supports a detailed analysis of phase behaviour and degradation dynamics, such as halide segregation (Fig. 4 and 5). Though this introduces some light–dark cycling and limits temporal resolution, it does not compromise the primary goal of perovskite optimization: ranking samples by performance and relative stability under accelerated aging conditions.

A key motivation for developing HITSTA was the desire to generate datasets with the granularity and reliability needed for machine-learning optimization campaigns. These require consistent evaluation of all samples using the same objective function between batches, which is difficult to achieve without automation. Manual methods, such as light-soaking with periodic UV-vis or PL measurements, will inevitably introduce variability in measurement timing, and possibly in ambient exposure. In addition, machine-learning algorithms typically explore both high-performing and poorly performing regions of parameter space simultaneously, resulting in batches with large variations in stability: some samples may degrade within hours, while others will remain stable for hundreds of hours. Reliable machine-learning therefore benefits from automated, frequent measurements to capture data on both ends of the stability spectrum, and to thereby build an accurate surrogate of the objective function. HITSTA provides such capability along with the option to perform significantly accelerated testing at elevated temperatures and high light intensities, allowing for rapid machine-guided iteration in high-dimensional spaces.

We believe that it is important to assess HITSTA in the context above: not as a substitute for high-precision UV-vis or PL-QY tools, but as a platform for reproducible, comparative assessments to drive optimization. Still, future upgrades could expand its scope. For example, adding bottom illumination under each sample would enable separate transmittance and reflectance measurements, complemented by replacing reflective enclosures with absorptive ones to reduce back-scatter. However, such configurations may be more sensitive to sample roughness,^22^ a challenge mitigated by HITSTA's current Ulbricht-style enclosure (Fig. 1a). For now, the combination of transflectance and PL proves highly effective for tracking degradation and ranking performance (Fig. 2 and 4–7).

A simple yet valuable enhancement for HITSTA would be mounting a camera on the measurement head to capture images during each cycle, enabling visual tracking of changes and offering additional diagnostic information over time.

## 4 Conclusions

Accelerating the optimization of halide perovskite films for both performance and stability remains a critical challenge that we have addressed through two complementary systems: HITSTA and ROSIE. Both systems are inexpensive (costing approximately €2300 and €1575 respectively in components and raw materials) and are assembled from a combination of off-the-shelf and 3D-printed components. Making them highly customizable and adaptable to various research needs.

HITSTA is a relatively simple yet robust platform built around a repurposed 3D printer. By operating at the film level, HITSTA potentially circumvents the additional steps and infrastructure required to fabricate and age full devices, offering a more agile approach to material development. A key innovation lies in its optical design, which enables sensitive, concurrent monitoring of sample absorption and photoluminescence through a single fiber head. With an integrated aging lamp, samples are shuttled automatically between measurements and aging, enabling almost uninterrupted, time-resolved stability assessments. This orchestrated interplay of characterization and aging not only reduces the labor burden on lab staff but also provides a nuanced understanding of material degradation dynamics.

Built around a hobbyist robotic arm and syringe pump, ROSIE achieves volumetric accuracy comparable to commercial systems. Other advantages include a relatively small footprint for glovebox integration (30 × 40 × 35 cm in its current implementation, *versus* 63 × 57 × 66 cm for the Opentrons OT-2, or 95 × 51 × 77 for the Hamilton Nimbus), and being fully customizable at the software level with flexible python scripting.

Together, HITSTA and ROSIE provide a powerful toolkit for accelerating the development of halide perovskite materials. By lowering the barriers to entry for high-throughput experimentation, these systems have the potential to drive rapid advancements in the field, ultimately contributing to the development of more efficient and stable perovskite-based devices.

## Author contributions

H. A. C. contributed to the conceptualization, investigation, methodology, software development, visualization, and writing of the original draft, as well as review and editing. D. A. J. was involved in the methodology and software development. N. F. contributed to the methodology. C. M. W. was responsible for the conceptualization, funding acquisition, methodology, resources, supervision, and writing – review and editing. C. B. provided supervision, resources and review.

## Conflicts of interest

There are no conflicts to declare.

## Supplementary Material

DD-004-D5DD00110B-s001

DD-004-D5DD00110B-s002

DD-004-D5DD00110B-s003

## Data Availability

The dataset for the showcase experiment is available at https://doi.org/10.5281/zenodo.14983975. STL files for ROSIE and HITSTA, along with example measurements from HITSTA, can be found at https://github.com/hilalaybikecan/AutoPVLab.git. Videos of ROSIE and HITSTA in operation are provided as Movies S1 and S2, respectively. HITSTA: system layout and rationale, description of white-light measurements, mode of operation; ROSIE: system description, summary of assembly, mode of operation, description of calibration and benchmarking procedures, discussion on solvent evaporation; discussion sections on data processing and assembly difficulty for both systems. See DOI: https://doi.org/10.1039/d5dd00110b.
