# Supplementary material for: Accelerating optimization of halide perovskites: two blueprints for automation
Source: Digit Discov. 2025 Aug 25;4(10):2804–15. doi: 10.1039/d5dd00110b (PMC12381630; doi:10.1039/d5dd00110b)
Supplement: DD-004-D5DD00110B-s001 [file DD-004-D5DD00110B-s001.pdf]

Supplementary Information (SI) for Digital Discovery.
This journal is © The Royal Society of Chemistry 2025

Supplementary Information

Accelerating Optimization of Halide Perovskites: Two Blueprints for Automation

Hilal Aybike Can,*[a] Daniel Anthony Jacobs,[b] Nicolas Fürst,[a] Christophe Ballif [a,b] and Christian Michael Wolff*[a]

1. HITSTA (High-Throughput Stability Testing Apparatus) 2
1.1 HITSTA – System layout and rationale: 2
1.2 HITSTA – White light measurements: 3
1.3 HITSTA – Mode of operation: 4
2. ROSIE (Robotic Operating System for Ink Engineering) 5
2.1 ROSIE – System description: 5
2.2 ROSIE – Summary of assembly: 5
2.3 ROSIE – Mode of operation: 6
2.4 ROSIE – Calibration and benchmarking: 7
2.5 ROSIE – Solvent evaporation: 7
3. ROSIE and HITSTA – Assembly difficulty and required expertise 8
4. ROSIE and HITSTA – Data processing 9
5. Supporting Figures 10
6. Supporting Tables 23

1. HITSTA (High-Throughput Stability Testing Apparatus)

1.1 HITSTA – System layout and rationale:

HITSTA is an optical characterization and aging platform based on a 3D printer (Creality Ender 3 Pro). Basing the system on a 3D printer jumpstarted the assembly by providing the functionality of a motorized stage and a heated sample-stage in one affordable purchase. The printer's microcontroller furthermore allows for easy control of both aspects via the broadcast of simple G-code strings over the COM interface. The Ender 3 in particular was chosen for being an affordable option with a relatively large build volume (220 x 220 x 250 mm), and for its simplicity, which smoothed the process of making modifications.

The first and primary modification needed to take the Ender 3 from a printer to an optical characterization platform was the design of a modified print-head, replacing the filament extruder with a mounting for optical fibers. Initially, three optical fibers were carried by the measurement head, two for "inputs" (white-light from a halogen lamp and a fiber-coupled laser) and one output fiber to the spectrometer. In this arrangement the white-light for transflectance measurements was directed down onto samples in a small spot (<2mm) before being transmitted to (and reflected from) mirrors placed behind each sample. Although a functional arrangement, we found that in this configuration HITSTA was quite sensitive to the effect of scattering by rough samples, since the tight white-light beam was then easily directed outside the collection cone of the measurement fiber. To rectify this situation, the second and current design iteration incorporated white-light LEDs within the measurement head illuminating samples from behind a diffuser, whilst the mirrors behind each sample were replaced with a matt-white reflective surface and enclosure, with the intended effect of increasing the collection of light scattered by samples.

HITSTA includes an aging lamp to apply high-intensity light stress to samples, with modifications ensuring consistent aging conditions. To achieve the movement of samples from the *measurement position* (under the modified print head) to an *aging position* under the lamp, the printer's belt-driven y-axis was extended with an aluminum rod. A small modification to the printer's Marlin firmware was necessary to accommodate the increase in y-axis travel. The sample holder is thereby able to move under the aging lamp at controlled intervals, enabling automated aging and measurement. Mirrors were positioned around the aging lamp (attached to the printer's frame) to direct light towards the samples with better homogeneity. Homogeneity was improved further by adding a plexiglass diffuser to the lamp.

For thermally accelerated aging, heat is provided by the 3D printer's built-in heated bed, which is capable of temperatures up to 110°C. To improve temperature uniformity across the sample bed, and reduce the thermalization time, a thermal pad was placed between the bed and the aluminium sample holder. A heat map of the temperature distribution across 49 sample positions on the holder shows only small variations (±4°C, Fig. S4d). Measurements with a contactless thermometer indicate that when underneath the aging lamp, the temperature becomes more uniform still, ensuring that all samples are subject to a closely comparable acceleration factor.

In terms of software, a Labview program was developed to orchestrate the delivery of commands to the 3D printer, data acquisition from the spectrometer, and switching of the various components (aging lamp, laser, and measurements LEDs) via a USB relay board. This Labview program is highly customized to our specified hardware and we therefore have no plans to publish it at this time, although we may choose to distribute a more generic version, or a template, in the future.

1.2 HITSTA – White light measurements:

The white light measurement system consists of an integrating enclosure that combines a sample backplate with a modified print-head. This enclosure houses LEDs for broadband illumination and an optical fiber bundle that connects to both a spectrometer and a blue laser. To ensure uniform coverage of the sample surface, we designed the integrating enclosure baffle to be slightly larger than the sample itself. An LED ring positioned at the top of the enclosure and capped with a diffuser provides uniform illumination across the entire sample area.

Our initial design using a fiber-coupled white-light lamp (with a small illumination spot on the sample) frequently resulted in transflectance values exceeding 1, as shown in Fig. S5c. Furthermore, we observed that the sub-bandgap spectral region evolved during aging, potentially misrepresenting the perovskite absorber's stability by altering the band-edge characteristics. By implementing an LED ring within the enclosure, we significantly reduced scattering-related changes below the bandgap (Fig. S5d). This improvement preserves the integrity of spectral regions near the bandgap, which are critical for accurate assessment of sample stability.

To further enhance measurement accuracy, we implemented two additional modifications. First, we performed all measurements from the glass side of the samples, which somewhat reduced the effects of surface roughening over time. Second, we repainted the white matte reflective back-surface before each experiment to maintain consistent measurement conditions and prevent background yellowing—a phenomenon that could result from either material release from the perovskite absorber or paint degradation under high light and temperature conditions. We verified the stability of our illumination conditions by monitoring the white-light spectrum over time, confirming no significant changes throughout the experiments. The normalized reflection spectra of glass and ITO substrates, presented in Fig. S5b, provide further explanation for the minor variations observed below the bandgap.

Stability measurements can be evaluated based on various spectral changes, including band-edge softening, the yellow-phase step at shorter wavelengths, and temporal variations in photoluminescence (PL) peak intensity. Fig. S7 details the calculations for the band-edge slope and yellow-phase step. These calculations were applied to each measurement over time for every sample, enabling a quantitative comparison of stability.

To determine the intensity of the aging lamp we first acquired a measurement of the lamp spectrum using our USB spectrometer, shown in Fig. S8a alongside the AM1.5G spectrum as a reference. To determine the scaling factor between the spectrometer measurement $m(\lambda)$ in arbitrary units and the aging lamp's true photon flux density $S(\lambda)$ (at the measurement bed, $S(\lambda) = a \cdot m(\lambda)$ for some constant a) the short-circuit current of a reference cell was measured on the bed and combined with its measured EQE such that

$$\begin{aligned} j_{sc}^{ref} &= \int S(\lambda) EQE^{ref}(\lambda)\, d\lambda \\ &= \int a \cdot m(\lambda) EQE^{ref}(\lambda)\, d\lambda \end{aligned}$$

i.e. $S(\lambda) = \frac{j_{sc}^{ref}}{\int m(\lambda) EQE^{ref}(\lambda)\, d\lambda} m(\lambda)$. To next determine the approximate illumination level as a function of perovskite bandgap (E_g) (Fig. S8c) we began by taking the measured EQE of a perovskite cell with a known bandgap of 1.51eV (Fig. S8b), denoted $EQE^{1.51eV}(\lambda)$, and scaling it as $EQE^{scaled}(E_g, \lambda) = EQE^{1.51eV}(\lambda \cdot \frac{Eg}{1.51eV})$. Next, the illumination level shown in Fig. S8c was computed based on the expected current for a cell with $EQE^{scaled}(E_g, \lambda)$ under the lamp spectrum $S(\lambda)$, and expressing this as a fraction of the current expected under AM1.5G, i.e.

$$I(E_g) = \frac{\int S(\lambda) EQE^{scaled}(E_g, \lambda)\, d\lambda}{\int AM1.5G(\lambda) EQE^{scaled}(E_g, \lambda)\, d\lambda}$$

1.3 HITSTA – Mode of operation:

A typical HITSTA experiment in our laboratory involves the fabrication of perovskite samples in a nitrogen glovebox specifically dedicated to spin-coating, after which samples are transferred using a sealed sample-box to another nitrogen glovebox containing the HITSTA platform. Operating through the glovebox, samples are then loaded into the sample positions manually. Underneath each sample is placed a disposable spacer glass, both to avoid scratching the sample and so that spacers can be replaced each experiment to limit cross-contamination.

In order to compute physically meaningful spectra in the analysis stage, the first two spaces on the sample bed of HITSTA are permanently occupied by a black absorptive back-sheet, simulating a fully absorbing sample, and an empty enclosure simulating a fully transparent sample. Measurements are conducted during each round at these positions to correct for background effects and as a crucial reference for the calculation of transflectance. By re-taking these measurements in every round we correct for possible drift of the illumination intensity in both the PL laser and white-light measurement LEDs.

In the simplest "one-shot characterization mode" the system moves through the following stages:

1. The sample bed is set to the requested measurement temperature and a stabilization period follows.
2. A single measurement cycle begins by measuring a "background" above each sample, i.e. by taking measurements with the spectrometer whilst both the measurement LEDs (in the modified print-head) and laser are switched off.
3. The white-light LEDs are switched on and a stabilization period follows.
4. White-light measurements are conducted by taking one or more acquisitions (averaging for noise reduction) above each sample position on the bed. The white-light LEDs are then switched off.
5. The blue laser is switched on and a stabilization period follows.
6. PL measurements are conducted by taking one or more acquisitions above each sample position on the bed. The laser is then switched off.
7. Raw data is automatically saved for external analysis, at which point transflectance and PL spectra are computed via appropriate background subtraction and normalization using the reference measurements.

In "aging-characterization mode" mode the system moves through the following stages in a continuous cycle:

1. A measurement round is triggered which follows the steps listed above for "one-shot mode".
2. The sample bed is set to the user-specified aging temperature (which may differ from the measurement temperature), and a stabilization period follows.
3. The aging lamp is switched on, and the printer's extended y-axis belt moves the sample-holder underneath the aging lamp.
4. After a user-specified (potentially variable) aging interval has elapsed, the aging lamp is switched off and the sample-holder is retracted back to the measurement position.
5. The sample bed is set to the requested measurement temperature and a stabilization period follows.
6. Return to 1.

We note that the aging intervals in "aging-characterization mode" can be pre-programmed arbitrarily, for example to conduct more frequent measurements at the beginning of an aging experiment, trending towards longer intervals as early "burn-in" phenomena (e.g. halide segregation) are surpassed.

2. ROSIE (Robotic Operating System for Ink Engineering)

2.1 ROSIE – System description:

At its core, ROSIE is the a combination of a modified 'uArm Swift Pro' robotic arm and a custom-designed syringe pump. The stock head of the uArm Swift Pro was modified according to the design of OpenLH (github.com/idc-milab/openlh): this involved 3D-printing a pipette tube holder, the latter being obtained by dismantling a spare manual pipette. The OpenLH pipette tube holder includes a mechanism that repurposes the actuator on the uArm into a release-mechanism for pipette tips. Tip attachment is performed simply by moving the arm onto the open end of the tip whilst it remains in the pipette box (see photographs below). The pipette box is replenished manually and held precisely in place using a simple 3d-printed enclosure in a fixed position relative to the arm.

The syringe pump and the robot arm are controlled from a master python script which sends serial commands to the syringe-pump Arduino (with CNC shield driving the syringe-pump stepper motor). The script employs a library provided by uArm (github.com/uArm-Developer/uArm-Python-SDK) to control the robot. This approach allows easy modification of the robot's behavior and extension to new applications such as drop-casting or spin-coating. The python control script and arduino code for the syringe-pump are shared on our github page (https://github.com/hilalaybikecan/AutoPVLab).

ROSIE's syringe pump was designed to provide precise control in the range of 1-200 µL. A stepper motor drives a precision gas-tight syringe, moving it forward or backward via a threaded mechanism to provide accurate aspiration and dispensing. The syringe is connected to a silicone tube, which links to the pipette tube held by the robotic arm (see Fig. S2a,b). To improve accuracy, the tube volume was reduced from our initial design and a smaller inner diameter tube was used, significantly enhancing liquid handling precision (Fig. S2d) and surpassing manual pipetting accuracy. Further refinements focused on minimizing error in liquid handling by controlling the air volume pushed or pulled through motor steps. Initial inaccuracies due to slack steps were addressed by replacing the spring coupling with a rigid one, and instead adding a spring to the connection point between the thread and mobile part (Fig. S2c). These changes reduced the relative error from 16% to 1.5% when handling 45 µL of solution (Fig. S2d). This value is lower than the 3% error observed with the lab's commercial manual pipettes (20-200 µL range), demonstrating that the liquid handling system now offers competitive precision suitable for most experiments.

2.2 ROSIE – Summary of assembly:

Robot arm: off-the-shelf uArm with modified head for holding pipette tips.

- The pipette holder is the assembly of a pipette tube taken from a 200uL manual pipette, and a 3D-printed holder designed by the OpenLH project.

Syringe pump:

- Main chassis of CNC-machined aluminium, design provided on the project's GitHub page (https://github.com/hilalaybikecan/AutoPVLab), held together with screws.
- A rigid shaft coupler connects the stepper-motor and the threaded rod.
- A flanged leadscrew nut rides on the threaded rod. The surface for actuating the syringe is expanded by attaching a 3D-printed part that effectively extends the leadscrew's flange, and rides on two fixed aluminium rods for extra stability.
- Two circular aluminium rods are used as railings to guide the pusher.
- The gas-tight syringe is mounted opposite the stepper with plunger attached to the pusher.
- A narrow diameter silicone tube attaches the syringe pump and the pipette head.

2.3 ROSIE – Mode of operation:

The following describes the typical operation of the ROSIE system:

1. The user prepares the stock vials to be mixed, and places these in the 3D-printed holder mounted at a fixed position on the optical breadboard (these positions are hard-coded in the python script). The user furnishes the pipette tray with the necessary number of pipette tips (positions also hard-coded).
2. The user supplies an excel sheet specifying the volume of each stock (spreadsheet column) to be dispensed into each well (spreadsheet row) of the well plate.
3. ROSIE treats each stock in sequence:
    I. First, a fresh pipette tip is taken from the pipette tray.
    II. Solution is drawn from the current stock and dispensed into all wells with a non-zero amount specified in the corresponding column of the spreadsheet, returning to take more solution from the stock as many times as necessary.
    III. When the current stock has been delivered to all wells specified in the sheet, the tip is dispensed into a bin area and the script moves to the next stock.
4. When ROSIE has finished dispensing all solutions, or before they are used, the user performs a mixing operation using a 12-channel multi-pipette (aspirating and dispensing the full volume several times for each well), which is typically completed in less than a minute. Due to ROSIE's relatively slow aspiration/dispensing speed and the time needed to refresh its pipette tip, such manual mixing with a multi-pipette is significantly quicker than having ROSIE mix the wells individually.

Depending on the type of experiment being performed and the solutions in use, cross-contamination between wells could be more or less of a concern. There are several options for users to address this issue on the software side. By setting a simple height variable, the user can decide whether solution should be dispensed "in air", i.e. with the active tip above the level of any solution already present in the well, or whether the tip should enter more deeply into the well to dispense "in solution". The former option prioritizes an avoidance of contamination, but can lead to inaccuracies when dispensing small volumes, as surface tension can limit the release of small droplets into the air. Conversely when dispensing into solution we attain a higher accuracy but suffer the risk of cross-contamination. In our experience performing many experiments with ROSIE and observing its operation at close quarters, it is extremely rare to observe residual solution adhering to the outside of the pipette tip, and when this occurs it amounts to only a very small quantity (a few uL at most, this being a few percent of the well volume). We estimate that the cross-contamination occurring via other mechanisms, such as the entry of solution into the tip via hydrostatic pressure, is likewise very small and a negligible component of our total experimental uncertainty.

To avoid cross-contamination without compromising dispensing accuracy ROSIE's python script could be trivially modified to take a new pipette tip after every dispense operation, although this might be somewhat costly and slower for large solution arrays.

2.4 ROSIE – Calibration and benchmarking:

Calibrations of ROSIE (determining the relation between stepper motor increments and volume dispensed), as well as accuracy and reliability benchmarks were conducted at various points during the system's development. For calibration, two key numbers had to be determined, namely the number of "slack" steps needed to tension the spring that couples the motor's motion to the syringe (relevant only during reversals of the syringe motion), and the translation factor between stepper motor increments and microlitres dispensed. These measurements were carried out by aspirating and dispensing volumes of DMF from a vial and measuring changes in weight on a sensitive self-calibrating microbalance (VWR). Some results of the benchmarking are shown in Fig. S2d, taken at various design stages for the isolated syringe pump. In Fig. S2 e,f we show calibration measurements of the fully assembled ROSIE platform, demonstrating a precision of ±0.6µL (random error, standard deviation from 24 dispense operations) in the range of 20-200µL. With smaller volumes of 5µL and 10µL we noted systematic under-delivery of approximately 0.3µL, which inflates the relative error in this range (Fig. S2f). We suspect that this systematic under-delivery of 0.3µL affects the entire volume range up to 200µL, and represents a small retention within the pipette tip that the syringe pump is unable to purge. As a future improvement of the ROSIE platform we may experiment with software compensations for this effect.

2.5 ROSIE – Solvent evaporation:

A certain amount of evaporation inevitably occurs from well plates during solution preparation with ROSIE, which will ultimately affect the accuracy of the prepared solutions. Measurements with DMF indicate an evaporation rate of approximately 2µL/h from each well at an ambient temperature of 22˚C inside an N_2 glovebox. This amounts to a roughly 1% change in concentration over the course of the preparation (assuming a typical well volume of 200uL and 1h running time). For DMSO similar measurements yield a smaller rate of 0.5µL/h. Neither of these values are therefore especially large contributions to the solution mixing error, however with more volatile solvents evaporation would become a problematic limitation on accuracy. This could be mitigated by increasing ROSIE's speed as discussed in the main manuscript, or by adding a Peltier-cooled bed for the well-plate to reduce evaporation rates.

3. ROSIE and HITSTA – Assembly difficulty and required expertise

Here we attempt to give a brief description of the technical expertise and time investment required to assemble ROSIE and HITSTA. We note at the outset that we have aimed to be transparent about the assembly process without providing full step-by-step instructions; however, we are open to providing additional guidance upon request. In our estimation, most research laboratories have personnel with the necessary skills to assemble both systems, as these are the same skills required for maintaining and repairing typical laboratory instrumentation: basic competence in electronics, mechanical assembly, and software operation. No highly specialized training in mechanical or electrical engineering is required.

Of the two systems, ROSIE is by a significant margin the simpler to assemble. The majority of parts are commercially available off-the-shelf; only the syringe pump chassis is custom, and this can be ordered directly from a contractor using the CAD files we supply on our github page. Assembly involves:

- Simple wiring of a stepper motor to its Arduino controller and driver shield.
- Basic familiarity with Python to operate the robotic arm and software.

With all parts in hand, ROSIE could feasibly be assembled and calibrated in a working day or two. We consider the level of difficulty commensurate with an undergraduate semester project in applied physical sciences or engineering.

HITSTA requires more involved modifications to the stock Ender 3 printer, particularly when the aging lamp capability is included, as for this the Y-axis must be extended to accommodate the lamp assembly. Installation and safe wiring of the power supplies for high-power LEDs require additional care. A minimal version of HITSTA, equipped only with white-light and PL measurement capabilities, is significantly easier to build. In this case, assembly would be limited to:

- Replacing the filament extruder with the 3D-printed optical head (our design is available on the github page), including attachment of the white-light measurement LEDs.
- Fabricating or ordering the sample plate and attachment to the printer bed.
- Making the optical connections for the laser (multi-core fiber to the head, connections to the spectrometer and laser).
- Completing basic wiring and implementing control software (e.g. LabVIEW or equivalent).

As such, we consider HITSTA's assembly to be more appropriately handled by a competent technician or experienced laboratory engineer with qualifications to safely work with high-power electronics.

4. ROSIE and HITSTA – Data processing

The data processing for a typical ROSIE + HITSTA experiment is handled by a suite of python scripts. These are responsible for translating the desired experiment in terms of a chemical sweep between specified stock solutions (e.g. $CsPbI_2Br$ and $CsPbI_3$), into a spreadsheet that is ingested by ROSIE's control script to perform the chemical mixing. Additional scripts perform the task of parsing the data files output by HITSTA, examples of which can be seen on our github page at github.com/hilalaybikecan/AutoPVLab/HITSTA/Examples. These analysis scripts compute primary quantities such as the PL and transflectance spectra, and then secondary quantities such as fits to the PL peak intensity over time or other metrics of performance and/or stability. Such metrics can then be used to produce plots, or output as objective functions to feed (for example) a Bayesian optimizer.

5. Supporting Figures

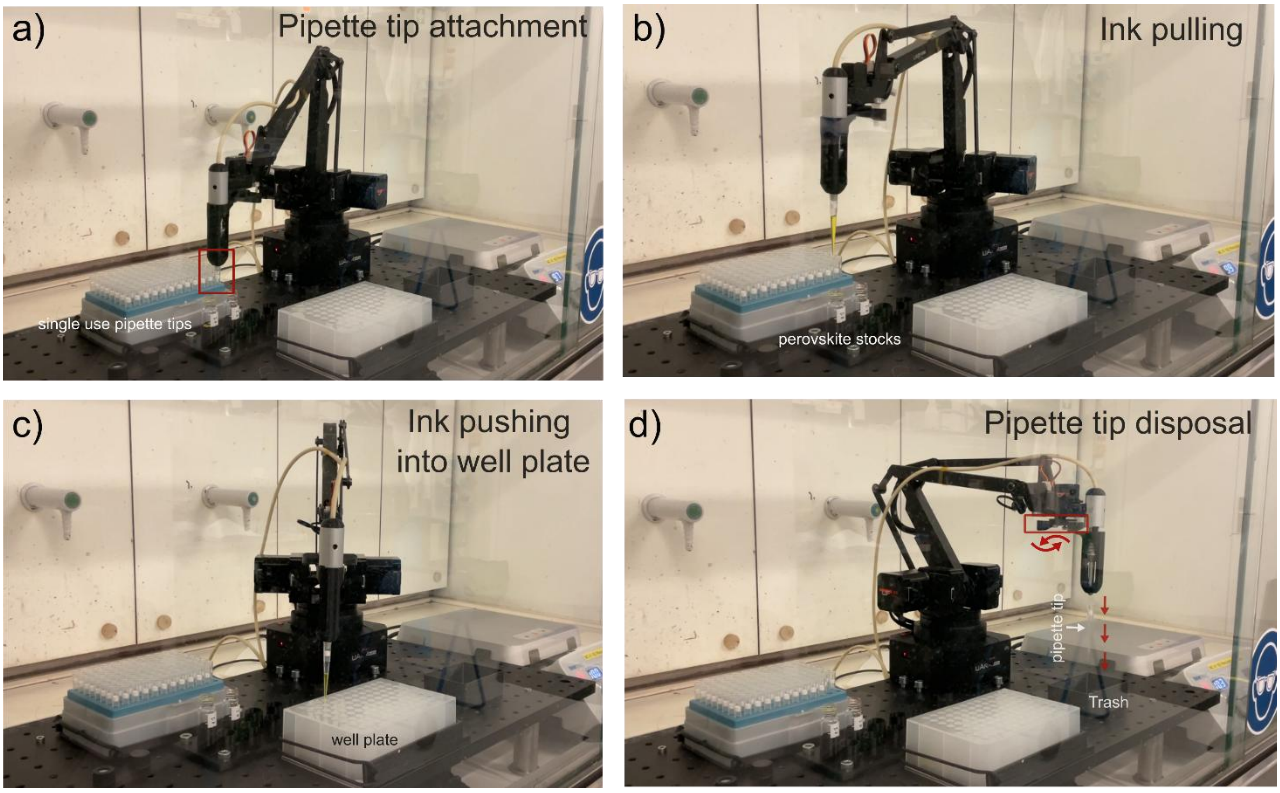

Fig. S1 Photographs of ROSIE's robotic arm at the moment of taking a fresh pipette tip from the holder (left image), and at the moment of dispensing the used tip into the designated bin (right image). The right image shows the modified head incorporating the pipette tube and holder as per the OpenLH design (github.com/idc-milab/openlh).

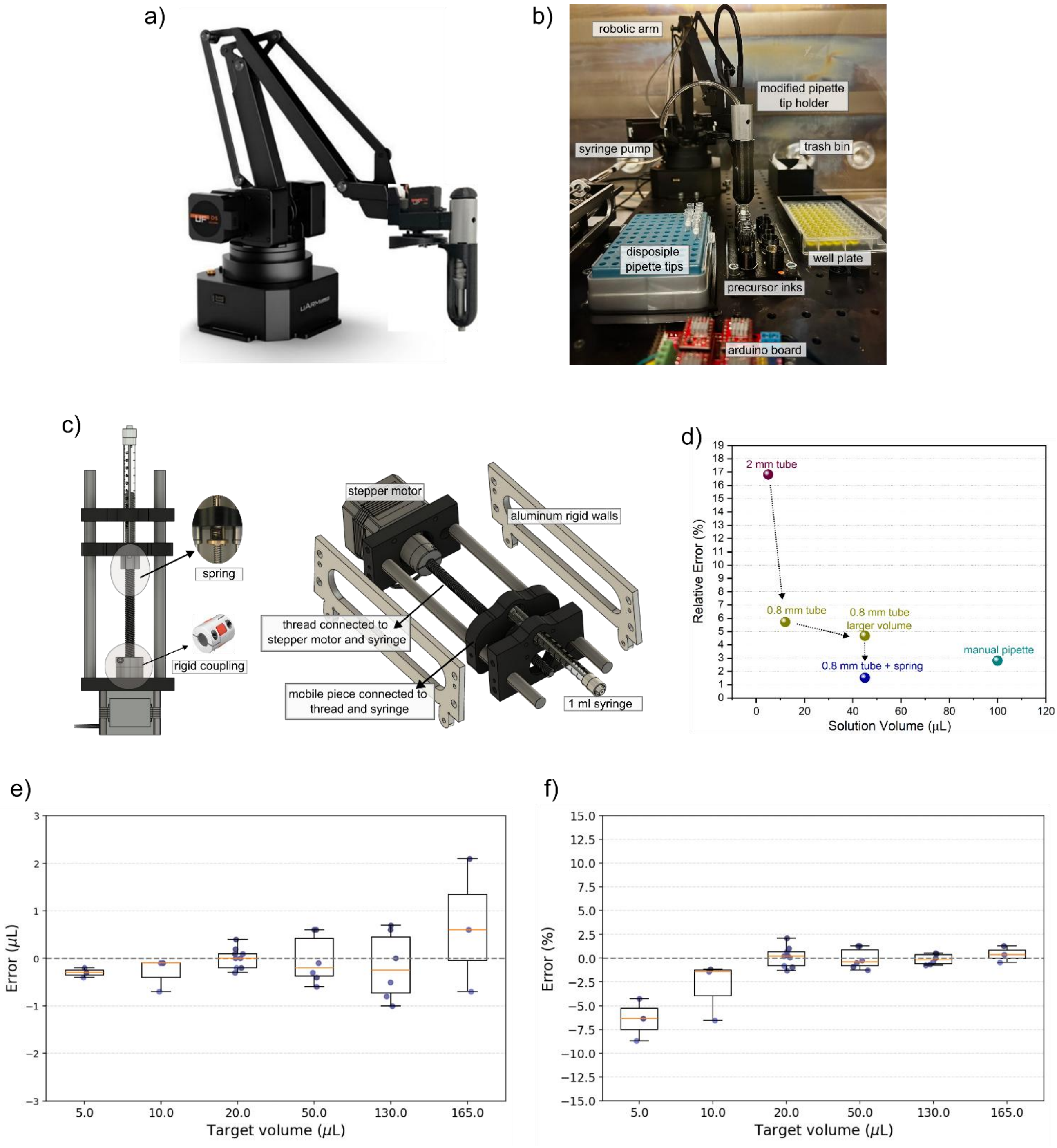

Fig. S2 a) uARM Swift Pro robotic arm with modified pipette tip holder, **b)** fully assembled ROSIE platform, **c)** Syringe pump 3D-design and components **d)** Relative error values during syringe pump optimization, showing the effect of design improvements (smaller diameter tube, adding the spring coupling) **e,f)** Volumetric error of the fully assembled system, as a function of the target volume in an absolute (**e**) and relative (**f**) sense.

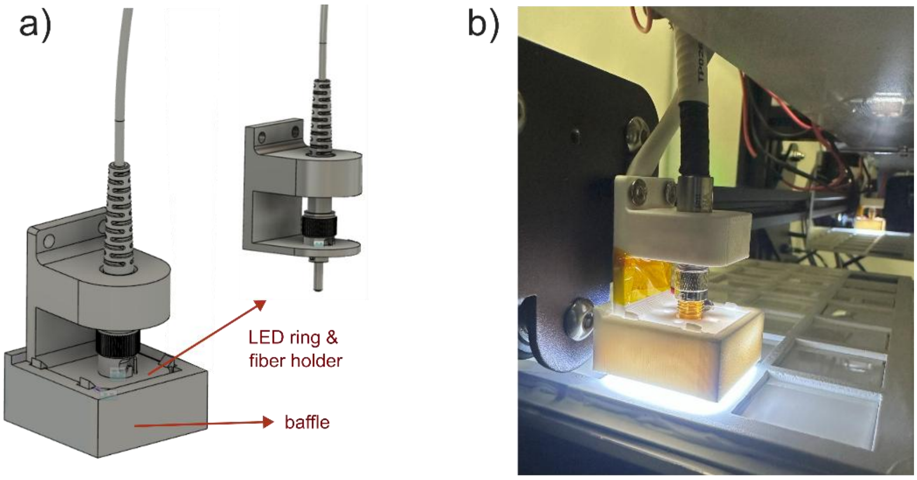

Fig. S3 a) The measurement head consists of a two-piece 3D-printed design, comprising a baffle and a fiber holder. An LED ring is positioned around the fiber, with a 1 mm thick PETG diffuser covering the LED ring to ensure uniform illumination. **b)** The assembled 3D-printed component is shown mounted on the HITSTA platform. The LEDs and laser are programmed to switch on and off at predefined intervals, enabling time-resolved measurements during the aging process.

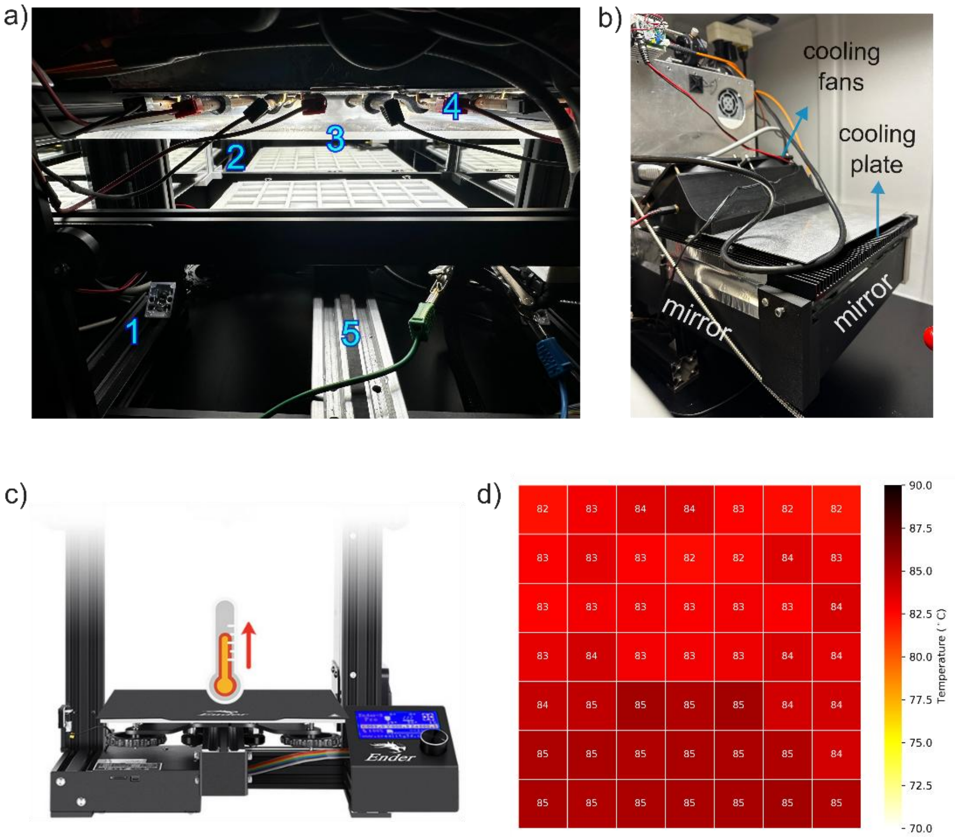

Fig. S4 a) Front view of the HITSTA layout, consisting of: (1) a 3D printer base, (2) mirrors positioned around the aging lamp, (3) a plexiglass diffuser for uniform light distribution during aging, (4) the power source for the aging lamp, and (5) a moving arm that transitions between the measurement and aging stages. **b)** Modified aging setup based on a 3D printer (rear view). **c,d)** The system also includes a heated bed to maintain controlled temperature conditions during aging. Temperature measurements across different areas of the bed are shown. The difference between the minimum and maximum temperatures decreases significantly under the aging lamp, ensuring uniform aging conditions for all samples

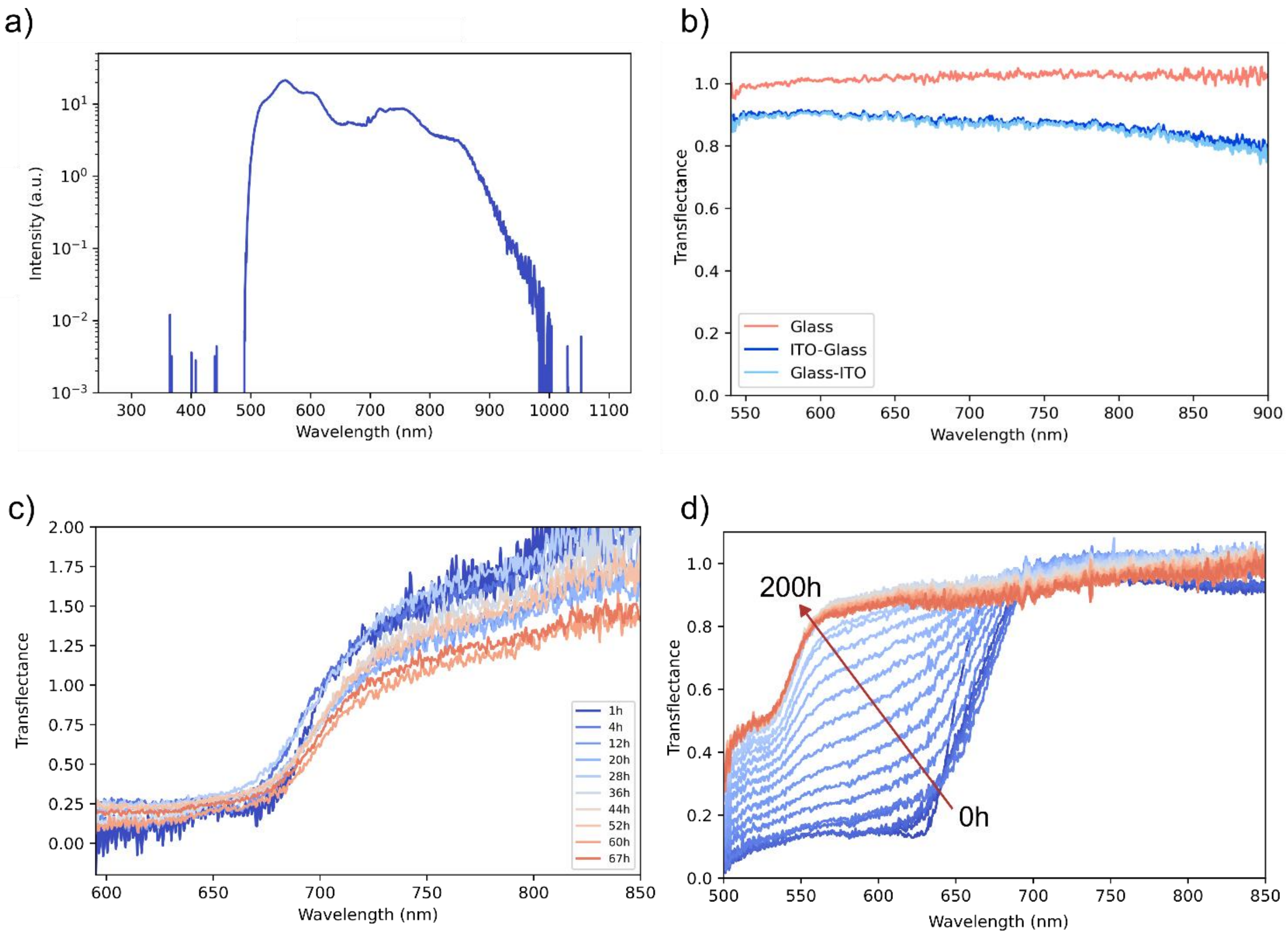

Fig. S5 Transflectance is calculated using white light data from a reference position on the sample plate (a reflective enclosure with no additional film), while dark measurements (with LEDs off) are recorded before each measurement for background subtraction. This latter step is crucial for long-term stability experiments, as it compensates for spectrometer drift due to temperature fluctuations in the glovebox. **a)** Broadband LED spectrum measured by HITSTA in an unoccupied sample enclosure. **b)** Transflectance measurements from uncoated glass and ITO substrates. **c)** Transflectance before incorporating the integrating enclosure, using a fiber-coupled white-light lamp with a small illumination spot on the sample. **d)** Transflectance after integrating enclosure was conceived and incorporated into HITSTA. Here wide-area illumination by the LED ring entails collecting data from the full sample area.

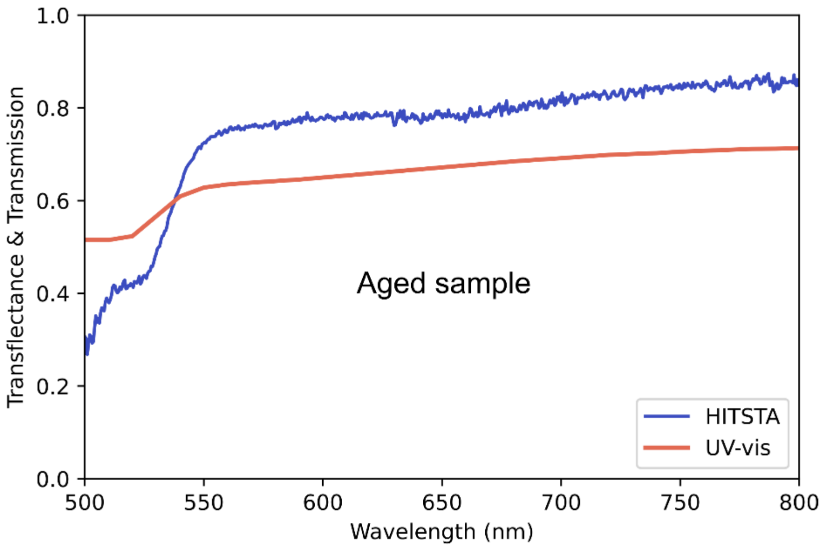

Fig. S6 Spectrum of a degraded sample measured with HITSTA and a UV-Vis spectrometer.

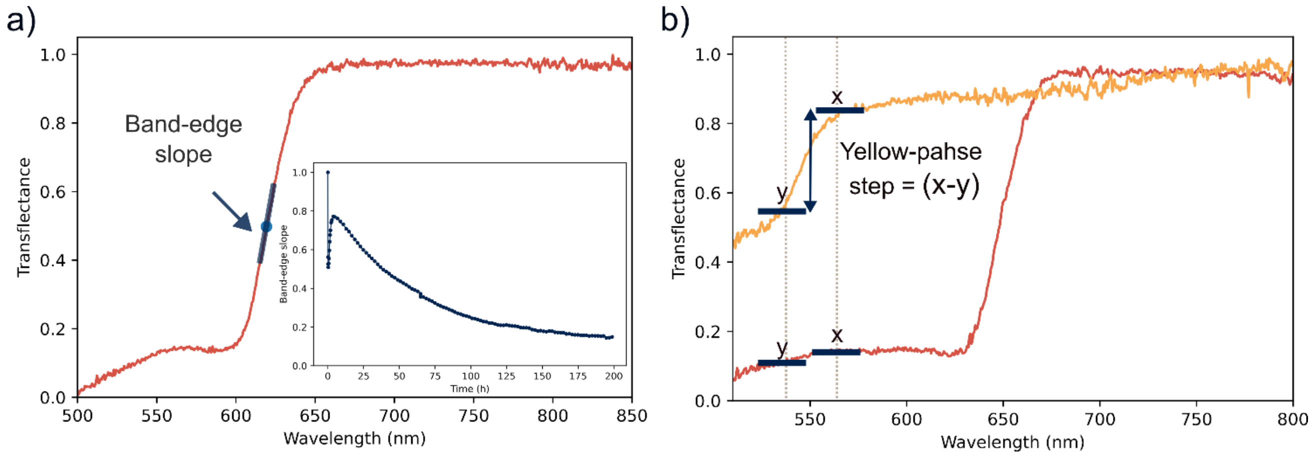

Fig. S7 a) The steepness at the band-edge is calculated by first identifying the midpoint of the band-edge, which is determined as the average of the minimum and maximum transflectance values. Next, a linear fit is applied in a 50nm interval extending to either side of this midpoint. A typical trend in the evolution of this slope is shown in the inset, with rapid changes (<1h) corresponding to halide segregation and slower changes indicating degradation to the yellow-phase. **b)** After tens to hundreds of hours, the band edge corresponding to the photoactive perovskite phase disappears, and a new band edge emerges around 540 nm. This transformation occurs gradually, and white-light data can effectively capture these subtle changes. The evolution of this band-edge at shorter wavelengths (yellow-phase step) is quantified by calculating the difference in transflectance values between 520 nm and 560 nm. Monitoring this difference over time provides insight into the rate of the yellow-phase transformation. Both the changes in band edge steepness and the yellow-phase transformation varied across samples, enabling us to rank the samples based on their stability and overall quality.

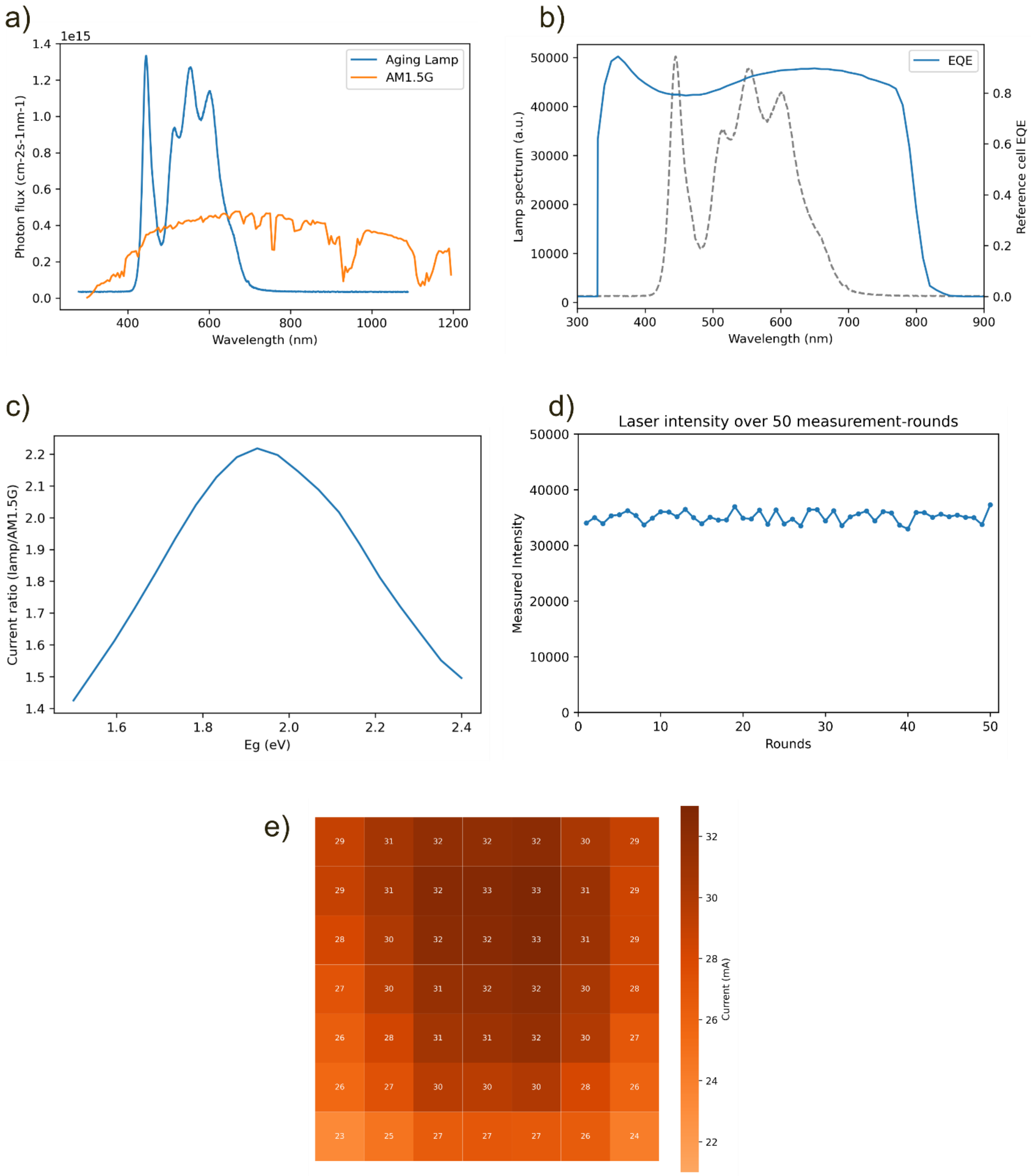

Fig. S8 a) Spectral photon flux comparison between an aging lamp source and the standard AM1.5G solar spectrum **b)** EQE of a perovskite cell with a known bandgap of 1.51 eV, with the lamp spectrum shown for comparison. **c)** The illumination-level computed based on the expected current for a cell with $EQE^{scaled}(E_g, \lambda)$ under the lamp spectrum $S(\lambda)$, and expressing this as a fraction of the current expected under AM1.5G. **d**) Measurements of HITSTA's PL laser diode (448nm) using the integrated spectrometer (the band-pass filters were removed for this measurement) over 50 consecutive rounds, showing less than 3% variation (standard error, σ/μ). **e)** Homogeneity of light intensity on the substrate holder, determined using the reference perovskite cell's current at each sample position on the sample-holder.

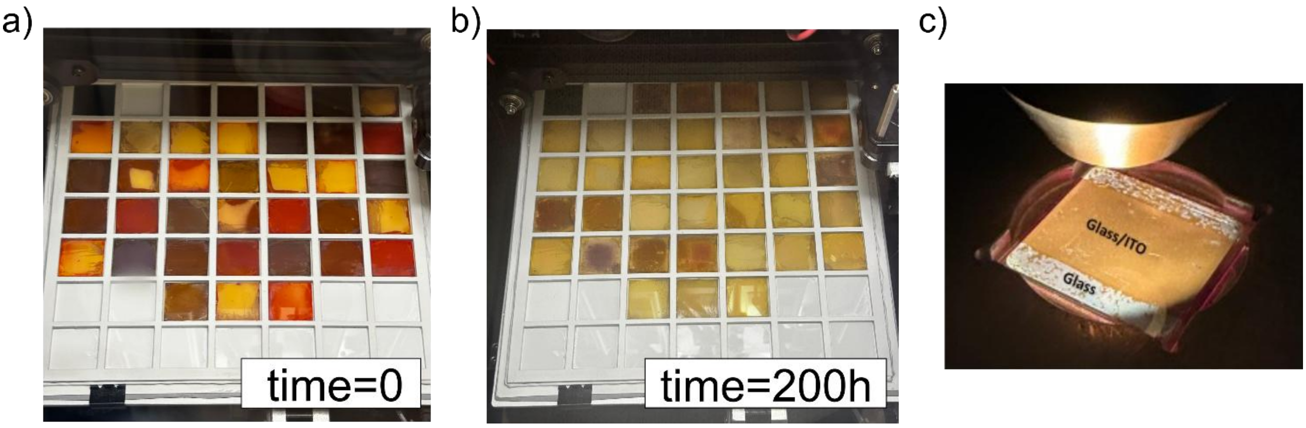

Fig. S9 a) Samples on the HITSTA sample holder immediately after spin coating. Some samples already presented with significant amounts of yellow-phase in this as-fabricated state. **b)** The same sample set after aging under 80®C and approx. 2.2-sun intensity for 200h. **c)** Photograph of an inorganic perovskite sample showing different degradation rates (here caused by prolonged air-exposure) in the glass-only and ITO-covered regions of a partial-area ITO substrate. This vividly demonstrates the importance of substrate choice on the resulting perovskite film properties.

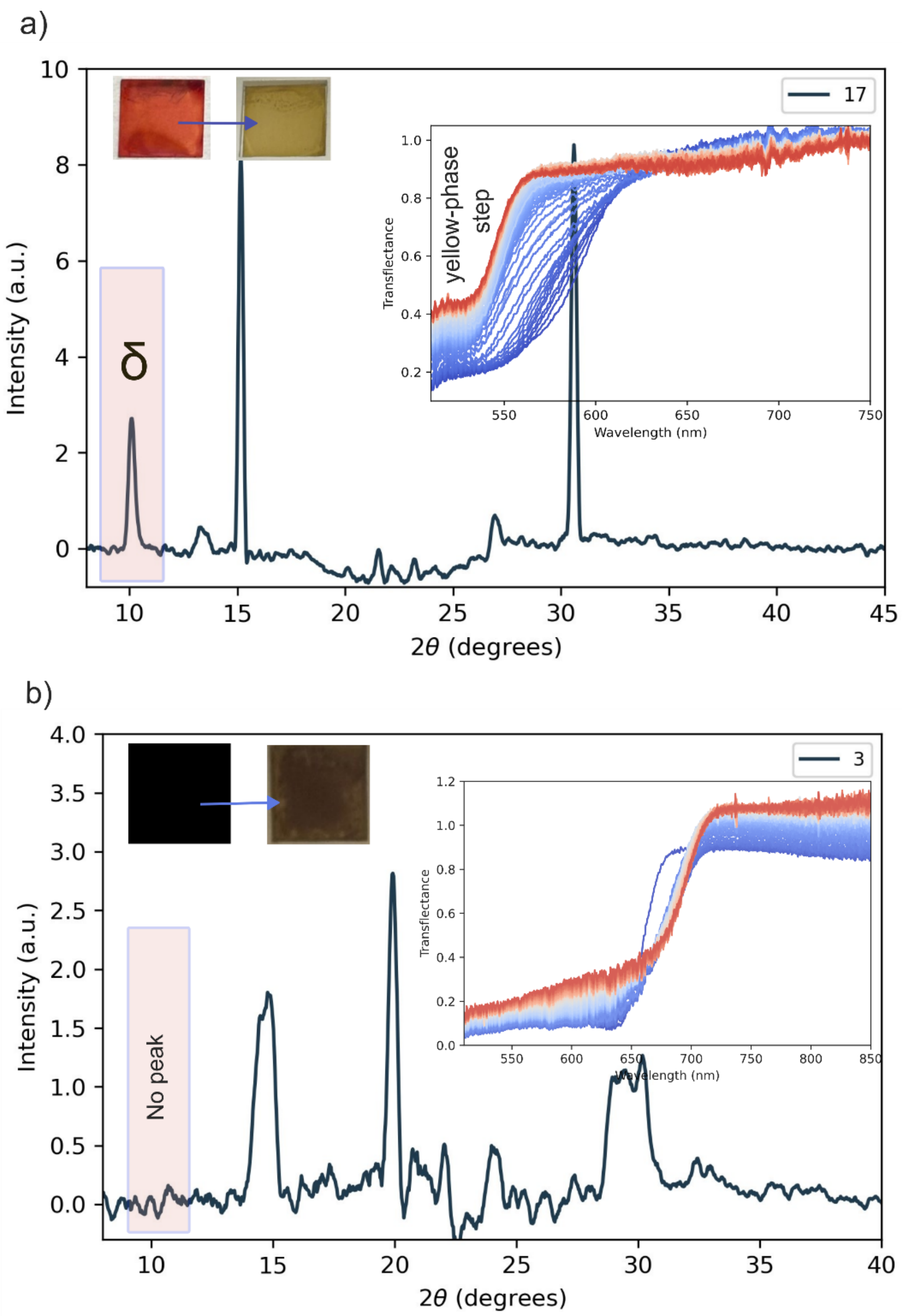

Fig. S10 XRD measurements of degraded samples from the paper's showcase experiment (aged for 80®C and approx. 2.2-sun intensity for 200h in HITSTA), showing in **a)** a sample ($Rb_{0.075}Cs_{0.925}PbI_{1.4}Br_{1.6}$ + 9.3% FACl) with a yellow appearance after aging (inset photos), a large step at 540nm in its transflectance spectrum, and a correspondingly large peak at 2θ = 10.0˚, shown by comparison with **b)** another sample ($CsPbI_2Br$ + 8% FACl) showing a relatively modest colour change after aging, a reduced step at 540nm in transflectance, and no detectable peak at 2θ = 10.0˚.

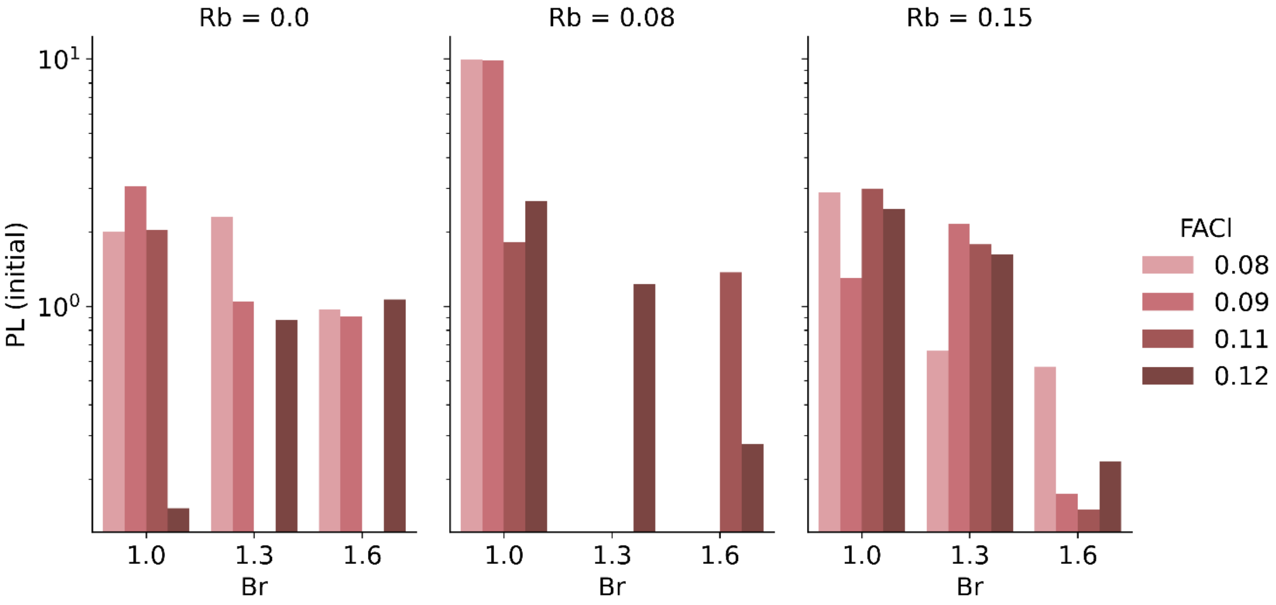

Fig. S11 Initial PL as a function of Br content for perovskites with varying Rb and FACl levels. Each panel shows a different Rb content; bar colors indicate FACl fraction.

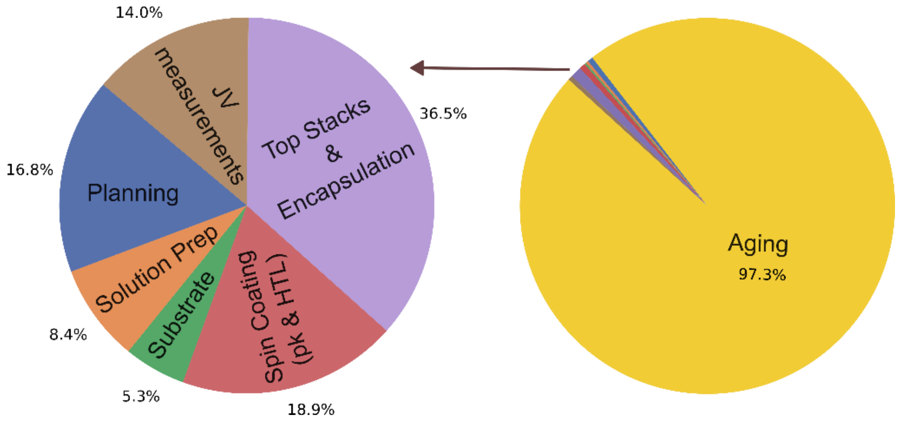

Fig. S12 Informal survey results conducted in our laboratory indicating the time required to execute a relatively standard perovskite experiment aimed at optimizing device efficiency (left) the corresponding breakdown for an experiment aimed at optimizing stability (right).

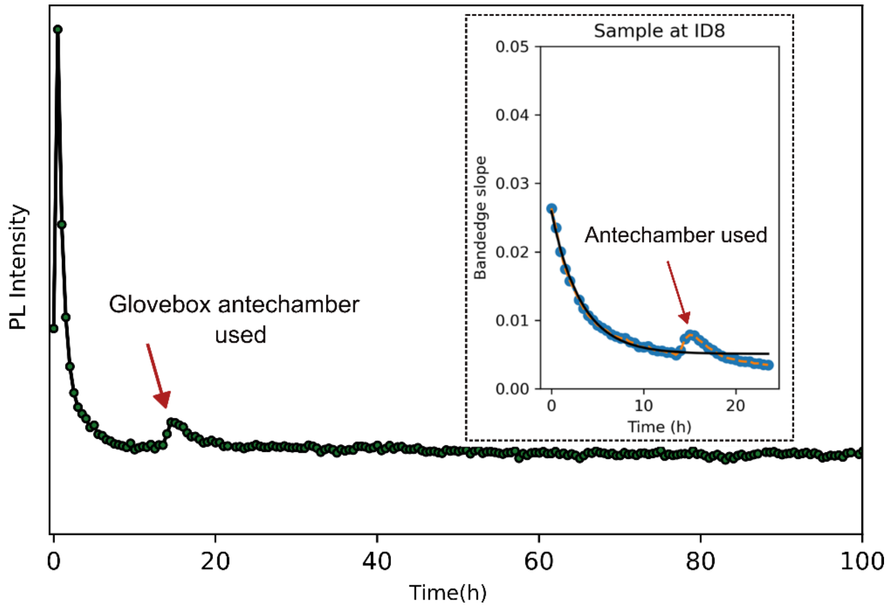

Fig. S13 Measurements are taken after glovebox antechamber is used. PL intensity and band-edge steepness shows some increase.

6. Supporting Tables

Table 1 Compositions were prepared in a well plate by ROSIE using eight stock solutions, as indicated in Fig.3 of the main text. During data analysis, each sample identified by its placement on the HITSTA sample holder.

Well plate	HITSTA location	Composition	FACl ratio
**A1**	3	$CsPbI_2Br$	8.0%
**A2**	4	$CsPbI_{1.7}Br_{1.3}$	8.0%
**A3**	5	$CsPbI_{1.4}Br_{1.6}$	8.0%
**A4**	6	$Rb_{0.075}Cs_{0.925}PbI_2Br$	8.0%
**A5**	7	$Rb_{0.075}Cs_{0.925}PbI_{1.7}Br_{1.3}$	8.0%
**A6**	8	$Rb_{0.075}Cs_{0.925}PbI_{1.4}Br_{1.6}$	8.0%
**A7**	9	$Rb_{0.15}Cs_{0.85}PbI_2Br$	8.0%
**A8**	10	$Rb_{0.15}Cs_{0.85}PbI_{1.7}Br_{1.3}$	8.0%
**A9**	11	$Rb_{0.15}Cs_{0.85}PbI_{1.4}Br_{1.6}$	8.0%
**B1**	12	$CsPbI_2Br$	9.3%
**B2**	13	$CsPbI_{1.7}Br_{1.3}$	9.3%
**B3**	14	$CsPbI_{1.4}Br_{1.6}$	9.3%
**B4**	15	$Rb_{0.075}Cs_{0.925}PbI_2Br$	9.3%
**B5**	16	$Rb_{0.075}Cs_{0.925}PbI_{1.7}Br_{1.3}$	9.3%
**B6**	17	$Rb_{0.075}Cs_{0.925}PbI_{1.4}Br_{1.6}$	9.3%
**B7**	18	$Rb_{0.15}Cs_{0.85}PbI_2Br$	9.3%
**B8**	19	$Rb_{0.15}Cs_{0.85}PbI_{1.7}Br_{1.3}$	9.3%
**B9**	20	$Rb_{0.15}Cs_{0.85}PbI_{1.4}Br_{1.6}$	9.3%
**C1**	21	$CsPbI_2Br$	10.6%
**C2**	22	$CsPbI_{1.7}Br_{1.3}$	10.6%
**C3**	23	$CsPbI_{1.4}Br_{1.6}$	10.6%
**C4**	24	$Rb_{0.075}Cs_{0.925}PbI_2Br$	10.6%
**C5**	25	$Rb_{0.075}Cs_{0.925}PbI_{1.7}Br_{1.3}$	10.6%
**C6**	26	$Rb_{0.075}Cs_{0.925}PbI_{1.4}Br_{1.6}$	10.6%
**C7**	27	$Rb_{0.15}Cs_{0.85}PbI_2Br$	10.6%
**C8**	28	$Rb_{0.15}Cs_{0.85}PbI_{1.7}Br_{1.3}$	10.6%
**C9**	29	$Rb_{0.15}Cs_{0.85}PbI_{1.4}Br_{1.6}$	10.6%
**D1**	30	$CsPbI_2Br$	12.0%
**D2**	31	$CsPbI_{1.7}Br_{1.3}$	12.0%
**D3**	32	$CsPbI_{1.4}Br_{1.6}$	12.0%
**D4**	33	$Rb_{0.075}Cs_{0.925}PbI_2Br$	12.0%
**D5**	34	$Rb_{0.075}Cs_{0.925}PbI_{1.7}Br_{1.3}$	12.0%
**D6**	35	$Rb_{0.075}Cs_{0.925}PbI_{1.4}Br_{1.6}$	12.0%
**D7**	38	$Rb_{0.15}Cs_{0.85}PbI_2Br$	12.0%
**D8**	39	$Rb_{0.15}Cs_{0.85}PbI_{1.7}Br_{1.3}$	12.0%
**D9**	40	$Rb_{0.15}Cs_{0.85}PbI_{1.4}Br_{1.6}$	12.0%

Table 2 ROSIE is controlled with a Python script which takes volumetric data for each well to be prepared as input. In this experiment a total of 220 µL solution was prepared using a grid-search combination of eight stock solutions.

Well No	Prec. 1	Prec. 2	Prec. 3	Prec. 4	Prec. 5	Prec. 6	Prec. 7	Prec. 8
**A1**	0.22	0	0	0	0	0	0	0
**A2**	0.11	0.11	0	0	0	0	0	0
**A3**	0	0.22	0	0	0	0	0	0
**A4**	0.11	0	0.11	0	0	0	0	0
**A5**	0	0.11	0.11	0	0	0	0	0
**A6**	0	0.11	0	0.11	0	0	0	0
**A7**	0	0	0.22	0	0	0	0	0
**A8**	0	0	0.11	0.11	0	0	0	0
**A9**	0	0	0	0.22	0	0	0	0
**B1**	0.14674	0	0	0	0.07326	0	0	0
**B2**	0.03674	0.11	0	0	0.07326	0	0	0
**B3**	0	0.14674	0	0	0	0.07326	0	0
**B4**	0.03674	0	0.11	0	0.07326	0	0	0
**B5**	0.07326	0.03674	0.03674	0	0	0	0	0.07326
**B6**	0	0.11	0	0.03674	0	0	0	0.07326
**B7**	0	0	0.14674	0	0	0	0.07326	0
**B8**	0	0	0.11	0.03674	0	0	0	0.07326
**B9**	0	0	0	0.14674	0	0	0	0.07326
**C1**	0.07326	0	0	0	0.14674	0	0	0
**C2**	0.07326	0	0	0	0.03674	0.11	0	0
**C3**	0	0.07326	0	0	0	0.14674	0	0
**C4**	0.07326	0	0	0	0.03674	0	0.11	0
**C5**	0.07326	0	0	0	0	0.03674	0.03674	0.07326
**C6**	0	0	0	0.07326	0	0.11	0	0.03674
**C7**	0	0	0.07326	0	0	0	0.14674	0
**C8**	0	0	0	0.07326	0	0	0.11	0.03674
**C9**	0	0	0	0.07326	0	0	0	0.14674
**D1**	0	0	0	0	0.22	0	0	0
**D2**	0	0	0	0	0.11	0.11	0	0
**D3**	0	0	0	0	0	0.22	0	0
**D4**	0	0	0	0	0.11	0	0.11	0
**D5**	0	0	0	0	0	0.11	0.11	0
**D6**	0	0	0	0	0	0.11	0	0.11
**D7**	0	0	0	0	0	0	0.22	0
**D8**	0	0	0	0	0	0	0.11	0.11
**D9**	0	0	0	0	0	0	0	0.22

Table 3 Approximate cost of ROSIE and HITSTA platforms

**HITSTA Components**	**Brand**	**Price (€)**
**3D Printer**	Creality Ender 3 Pro	200
**Spectrometer**	Ossila	1458
**Aging Lamp LEDs**	V-TAC	154
**High power broad band LED**	Roithner Lasertechnik	73.12
**Laser**	Insaneware	165
**Longpass colored glass filters**	Thorlabs	40
**Optical Fiber**	Thorlabs	424
**Thermal Pad**	Arctic	9.5
**Mirrors**		5
**Plexiglass diffuser**		5
**White Paint**		10
**3D printed pieces**		<10
**Cooling fans**		20
**AC-DC single output LED driver**	Mean Well	152
**LED radiator**		50
**Total**		**2765.62**
**ROSIE Components**	**Brand**	**Price (€)**
**Robotic arm**	uArm Swift Pro	933
**Platinum-cured silicone tube**		<1
**Longlife gastight 1mL syringe**	Hamilton	160
**Well plate**		2
**3D printed pieces**	<10	
**Aluminum optical breadboard**	Thorlabs	195
**Linear threaded rod (8mm)**		20
**Stepper motor**		15
**Arduino**	Arduino Uno R3	24
**CNC machined aluminum**		30
**Total**		1379
